# Ruthenium ion catalysed C–C bond activation in lignin model compounds – towards lignin depolymerisation†

**DOI:** 10.1039/d3cy00076a

**Published:** 2023-08-25

**Authors:** Susana Guadix-Montero, Mala A. Sainna, Jiangpeiyun Jin, Jack Reynolds, W. Graham Forsythe, Gary N. Sheldrake, David Willock, Meenakshisundaram Sankar

**Affiliations:** a Cardiff Catalysis Institute, School of Chemistry, Cardiff University Cardiff CF10 3AT UK sankar@cardiff.ac.uk +44 (0)2920 874 030 +44 (0)29 2087 5748; b School of Chemistry and Chemical Engineering, Queens University Belfast David Keir Building, Stranmillis Road Belfast BT9 5AG Northern Ireland UK

## Abstract

Lignin is the most abundant renewable feedstock to produce aromatic chemicals, however its depolymerisation involves the breaking of several C–O and C–C inter-unit linkages that connect smaller aromatic units that are present in lignin. Several strategies have been reported for the cleavage of the C–O inter-unit linkages in lignin. However, till today, only a few methodologies have been reported for the effective breaking or the conversion of the recalcitrant C–C inter unit linkages in lignin. Here we report the ruthenium ion catalysed oxidative methodology as an effective system to activate or convert the most recalcitrant inter unit linkages such as β-5 and 5–5′ present in lignin. Initially, we used biphenyl as a model compound to study the effectiveness of the RICO methodology to activate the 5–5′ C–C linkage. After 4 h reaction at 22 °C, we achieved a 30% conversion with 75% selectivity towards benzoic acid and phenyl glyoxal as the minor product. To the best of our knowledge this is the first ever oxidative activation of the C–C bond that connects the two phenyl rings in biphenyl. DFT calculation revealed that the RuO_4_ forms a [3 + 2] adduct with one of the aromatic C–C bonds resulting in the opening of the phenyl ring. Biphenyl conversion could be increased by increasing the amount of oxidant; however, this is accompanied by a reduction in the carbon balance because of the formation of CO_2_ and other unknown products. We extended this RICO methodology for the oxidative depolymerisation of lignin model hexamer containing β-5, 5–5′ and β-O-4 linkages. Qualitative and quantitative analyses of the reaction mixture were done using ^1^H, ^13^C NMR spectroscopy methods along with GC-MS and Gel Permeation Chromatographic (GPC) methods. Advanced 2D NMR spectroscopic methods such as HSQC, HMBC and ^31^P NMR spectroscopy after phosphitylation of the mixture were employed to quantitatively analyse the conversion of the β-5, 5–5′ and β-O-4 linkages and to identify the products. After 30 min, >90% of the 5–5′ and linkages and >80% of the β-5′ are converted with this methodology. This is the first report on the conversion of the 5–5′ linkage in lignin model hexamer.

## Abstract 2

Ruthenium ion catalyzed oxidative depolymerisation of lignin model hexamer.

## Graphical abstract

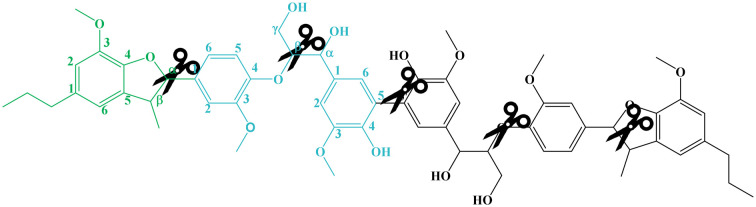

## Introduction

**Fig. 1:**
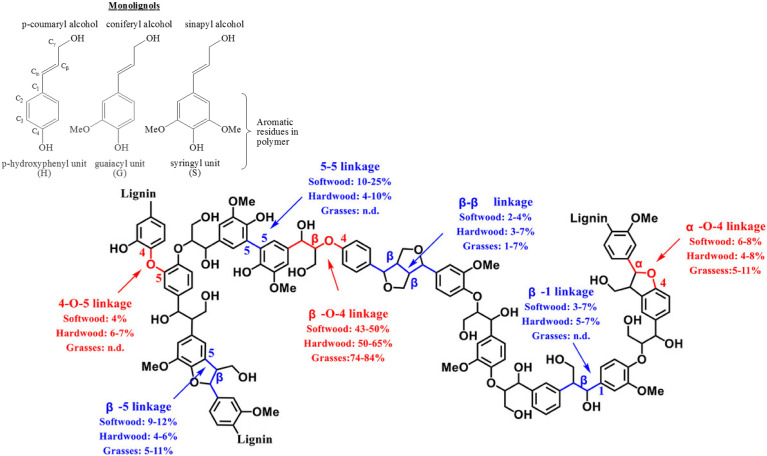
Representative structure of a typical lignin with different inter-unit linkages marked. Reproduced with permission from ref. 7. Copyright 2020, John Wiley and Sons.

Lignin, one of the three major components of lignocellulosic biomass, is a cross-linked amorphous phenolic biopolymer that gives plants their structural strength and shape.^1^ It contains highly functionalised aromatic units that makes them a potential renewable and sustainable feedstock to produce aromatics and has the potential to replace fossil-fuel based feedstock in producing fuel and base chemicals.^2^ Lignin constitutes approximately 30% of the mass and 40% of the energy content of lignocellulosic biomass. After cellulose, lignin is the most abundant carbon source on earth.^3^ Approximately 100 million tonnes of Kraft lignin is produced per year, within which approximately 70 million tonnes of lignin comes from paper and pulping industries.^4^ Lignins obtained *via* the commercial delignification processes are called as technical lignin and contain higher proportion of the C–C inter-unit linkages compared to the native lignins present in plants. Hence, to valorise lignin, it is crucial to break both the C–O and C–C inter unit linkages, to make biorefining economically viable and sustainable. The structure of lignin consists of three basic sub-units (*p*-hydroxyphenyl, guaiacyl, and syringyl, which are formed from the three monolignols *p*-coumaryl, coniferyl, and sinapyl alcohols respectively) that are linked by different kinds of C–O and C–C inter-unit linkages.^1,5^Fig. 1 shows different kinds of inter-unit linkages, structures of monolignols present in different lignins.^6^

These inter-unit linkages must be broken to obtain aromatic compounds from lignins, hence it is an important challenge for chemists. Depending on the biomass, 25–34% of the inter-unit linkages are C–C linkages and they are more recalcitrant compared to the C–O inter-unit linkages.^1,8^ The bond dissociation energies of C–C and C–O linkages are 226–494 kJ mol^−1^ and 209–348 kJ mol^−1^ respectively.^9–11^ Consequently, most of the reported valorisation strategies focus on the cleavage of C–O linkages resulting into *ca.* 60 wt% of un-valorised lignin fraction having predominantly complex C–C inter-unit linkages.^10^ Hence, developing a catalytic methodology for the effective cleavage of the recalcitrant C–C inter-unit linkages in lignin is an important and challenging problem. Oxidative, reductive, redox neutral, microwave assisted, photocatalytic, biocatalytic routes have been employed, with limited success, for the cleavage of C–C inter unit linkages.^10^ Among these, oxidative cleavage is more promising because it produces value-added aromatics having carbonyl groups.

**Fig. 2:**
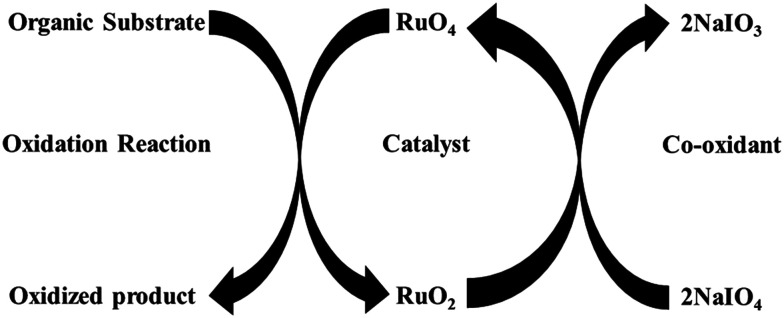
Schematic representation of ruthenium ion catalysed oxidation of organic compounds.

The seminal discovery of using ruthenium tetroxide (RuO_4_), instead of the more toxic OsO_4_, for the oxidation of organic substrates, by Djerassi in 1953, opened a new methodology to oxidize organic compounds.^12^ This was later named as ruthenium ion catalysed oxidation (RICO) where the active species (RuO_4_) is generated *in situ* by the combination of ruthenium ions and an oxidizing agent (NaIO_4_). During this oxidation reaction, Ru(viii) oxidizes the organic substrate to form Ru(iv), which is oxidized back to Ru(viii) by a co-oxidant such as NaIO_4_ (Fig. 2) and this cycle continues until all the substrate is oxidized.^13^

Typically, in this system, RuCl_3_·*x*H_2_O is used as the ruthenium source and NaIO_4_ as the oxidant to generate RuO_4_*in situ* to oxidize organic compounds in a biphasic mixture of dichloromethane and water.^14^ These oxidation reactions are fast and unselective because of the high reactivity of the oxidant, RuO_4_.^13^ Sharpless and co-workers improved the selectivity and stability of the catalytic system by adding acetonitrile in the solvent mixture.^15^ After that, RICO has been found to be effective the dihydroxylation of olefins, dehydrogenation of alcohols, amines, oxidative cleavage of double and triple bonds, oxidation of saturated hydrocarbons and aromatic hydrocarbons.^13,16^ The high reactivity of RICO chemistry has been exploited in the structural characterisation of coal, bitumen and polyaromatic hydrocarbons.^17^ RuO_4_ is used to completely oxidize the aromatic rings to carboxylic acid leaving the aliphatic groups intact. Recently, Nowicka *et al.* reported strategies to tune the selectivity of this reaction by changing the solvent system.^18^

The mechanism of the oxidation of olefins and polyaromatics by RuO_4_ has been addressed in a number of computational studies. Frenking and co-workers used the hybrid B3LYP functional and ethene as a model olefin to show that the initial co-ordination of substrate is *via* a five membered metallocycle in a [3 + 2] addition.^19^ This lowers the bond order of the alkene and reduces the metal centre from Ru^8+^ to Ru^6+^. Nowicka *et al.* extended this idea to polyaromatic hydrocarbons, such as pyrene, and demonstrated that the regioselectivity could be understood in terms of the formation of the most energetically favourable [3 + 2] regioisomer.^20^ These calculations also showed that the barrier to C–C bond cleavage in the metallocycle is lowered considerably if the Ru^6+^ metal centre is first reoxidised to Ru^8+^.^20^

Inspired by these developments, we employed the RICO chemistry for the oxidative activation or conversion of the most recalcitrant C–C inter-unit linkages found in lignin: the 5–5′ bond linking two aromatic rings (BDE 481–494 kJ mol^−1^).^1,9^ Here, we report the oxidative activation of the 5–5′ C–C linkage in biphenyl, which has one of the strongest C–C bonds (linking two phenyl groups) among all the lignin model compounds to benzoic acid. Though the 5–5′ C–C bond is still present in benzoic, it is much weaker and benzoic acid can be easily decarboxylated to benzene. Computational methods were employed to reveal the mechanism of the oxidative activation of this 5–5′(C–C) bond in biphenyl. We further extended this methodology for the oxidative depolymerisation of a more complex lignin model oligomer (named as hexamer) which has several inter-unit linkages (β-O-4′, 5–5′ and β-5′) that are typically present in real lignin. Our hypothesis is that the catalytic system that can convert the most recalcitrant 5–5′ linkage will be able to break all other linkages.

## Experimental

Materials: RuCl_3_·*x*H_2_O (>99.9%, Sigma Aldrich, certificate analysis 41.9%), NaIO_4_ (>99%, Sigma Aldrich), Na_2_SO_3_ (>98%, Sigma Aldrich), biphenyl (>99.5%, Sigma Aldrich). All the solvents were used without any further purification steps.

The ruthenium ion catalysed oxidation (RICO) reaction was carried out in a 50 mL round bottom flask fitted with a condenser, under vigorous stirring at 22 °C (room temperature). Water–acetonitrile mixture was used in all the mono-phasic reactions, where the substrate : oxidant : catalyst molar ratio was kept at 1 : 8 : 0.1 at the start. In a typical reaction, the glass reactor was charged with requisite amount of the substrate (0.164 mmol) and the solvent acetonitrile (20 mL) at 22 °C. To this solution, an aqueous solution of NaIO_4_ (280 mg, 1.312 mmol dissolved in 10 mL of deionized H_2_O) was added and stirred for 1 min (stirring speed: 500 rpm). Then solid RuCl_3_·*x*H_2_O (2.5 mg, 0.012 mmol) was added to the above mixture and this point is considered as the start of the reaction. To make the addition of catalyst precursor easier, we used an aqueous stock solution of RuCl_3_·*x*H_2_O, analysed by ICP (7.178 mg ml^−1^). After specific reaction time, an aliquot of the reaction mixture was taken out and quenched immediately by the addition an aqueous solution of Na_2_SO_3_. The number of moles of Na_2_SO_3_ was equal to the number of moles of the oxidant used in the reaction.

Depending on the substrate, the solvent in the quenched aliquots was removed *via* either freeze drying or in vacuum for qualitative analyses. The resultant solid was dissolved in 0.8 mL of d^6^-acetone and 0.088 mL of CDCl_3_ and then analysed by NMR spectroscopy (Bruker DPX 500 MHz instrument equipped with a 5 mm autotune broad band probe). Water suppressed NMR spectra were acquired over 32 scans using the Bruker pulse sequence *zgpr*. A power level of 53.5 db was employed for water pre-saturation, in conjunction with a relaxation delay of 5 s and an acquisition time of 1.638 s. Detailed methodology for the quantitative analyses of the reaction mixture, using NMR is provided in the ESI† including calibration plots (Fig. S1 and S2) and response factors (Table S1) used in the analyses.

GC-MS was used for the qualitative identification of some of the products from the reaction mixture. All samples were prepared by dissolving the concentrated reaction mixture in ethyl acetate (1 mL) and then syringe filtered (0.45 μm PTFE filter) and poured into a vial. The samples were analysed by Waters GCT premier instrument fitted with an Agilent HP-5MS column using He as the carrier gas.

For quantitative analyses, using HPLC, organic compounds in the reaction mixture were removed from the inorganic metal ions using a ternary mixed-solvent solution of water–acetonitrile–ethyl acetate.^21^ After quenching the reaction mixture with sodium sulphite, the solid materials formed during the reaction were filtered and washed with sodium hydroxide, in order to convert the carboxylic acid products (benzoic acid is sparingly soluble in water at room temperature) into their sodium salts. Diluted hydrochloric acid was then used to acidify this solution to convert the salt back to carboxylic acid prior to extraction using ethyl acetate. This ethyl acetate layer was used for all the quantitative analyses.

A control experiment was performed to confirm the quantitative extraction of all organic compounds after 4 extractions using 15 mL of ethyl acetate and the absence of any organic compounds in the aqueous layer. Inductively coupled plasma (ICP) [Agilent Technologies 7900 ICP-MS system, fitted with an Agilent integrated autosampler] analyses of both aqueous phases confirmed that sodium and ruthenium ions remain in the aqueous layer (ESI† Table S1 shows the concentration of metal ions in both aqueous and organic phases after extractions). HPLC (Agilent 1200 series fitted with a Diode Array and UV detectors) fitted with a Poroshell 120 SB-C18 4.6 × 150 mm, 2.7 μm column was used to analyse the organic and aqueous layers, after the post-reaction protocols, to quantify the efficiency of the extractions. For this control experiment, an aqueous mixture of biphenyl and benzoic acid was extracted using this protocol and after 4 extractions, the aqueous and the organic layers were analysed by HPLC. The data (ESI,† Fig. S3) shows that majority of the organic compounds were extracted in the organic layer in the first extraction itself and quantitative extraction was achieved after 4 extractions. Control experiments with oxidant alone without any Ru catalyst was performed using hexamer as the substrate at 22 °C for 16 h and the NMR spectra of the product mixture is provided in the ESI† (Fig. S4) which shows little or no catalytic activity.

We apply density functional theory with the hybrid B3LYP functionals to calculate energies of intermediates and transition states using the Gaussian09 package.^22^ The formation energy, *E*_f_, for [3 + 2] adducts was estimated using the relation:*E*_f_ = *E*_[3+2]_ − *E*_RuO_4__ − *E*_bip_where *E*_[3+2]_ is the energy of a given [3 + 2] adduct, *E*_RuO_4__ the energy of the RuO_4_ oxidant and *E*_bip_ the energy of biphenyl in isolation. All structures were fully optimised before calculation of the formation energy. In all calculations a consistent functional and basis set is used for all three calculations. This definition of *E*_f_ implies that a negative value is indicative of favourable complex formation.

6-311+G(d,p) atom centred basis functions were used on all elements aside from Ru for the calculation of *E*_f_ values. For calculations of chemical reaction intermediates and transition states the basis set was lowered to 6-31G(d,p). For Ru the LANL2DZ basis functions and corresponding effective core potential were employed throughout. A check of basis set superposition error (BSSE) using the counterpoise correction approach showed that the magnitude of *E*_f_ is underestimated by around 10% due to BSSE.

The [3 + 2] complex has a d^2^ configuration, all adducts were calculated in the singlet and triplet states. The singlet state was always found to be at least 30 kJ mol^−1^ more stable than the triplet and so only singlet energies are reported.

Transition states for the C–C bond cleavage in [3 + 2] intermediates was sought by a series of constrained optimisations with the C–C distance increased in steps of 0.1 Å. The maximum from the bond scan was then used in a frequency calculation and an eigenvector follower method used to further refine the energy maximum along the reaction co-ordinate. The transition state was confirmed by a further frequency calculation to confirm a single negative mode which was visualised to check it corresponded to C–C scission.

## Results and discussion

### Oxidation of biphenyl

**Scheme 1:**
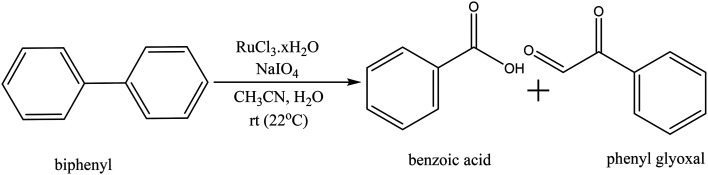
Ruthenium ion catalysed oxidation of biphenyl to benzoic acid.

Biphenyl is one of the model compounds that represents the 5–5′ linkage in lignin. It represents one of the strongest interunit linkages present in lignin (BDE 481–494 kJ mol^−1^).^1,9^ Hence, it was chosen for the initial studies to oxidatively break the C–C bond. Ruthenium ion catalysed oxidation at 22 °C of biphenyl resulted in benzoic acid as the major product along with minor quantities of phenylglyoxal, toluene, *o*-xylene, benzaldehyde cinnamyl alcohol and cinnamaldehyde. These products were identified *via* the GC-MS studies of the products mixture (ESI,† Table S3). This is an indicative list because GC-MS has identified all the compounds including solvent impurities. HPLC was employed for the quantitative analyses of the products in the reaction mixture. Calibrations were performed for biphenyl, benzoic acid, and phenyl glyoxal only to reduce the complexity of the analysis (Scheme 1). A few unidentified products were seen in the HPLC, however, they were minor quantities. A typical HPLC chromatogram is presented in the ESI† (Fig. S2). The carbon-balance of the reaction mixture was always calculated to indicate the quantity of these unknown products. The carbon balance was always *ca.* >80% unless reported otherwise. Although the conversion and selectivity values were calculated using calibration curves and response factors, the error in this value is higher than a typical analysis by HPLC because of the combination of experimental errors during extraction, post synthesis work-up, and unidentified products.

**Fig. 3:**
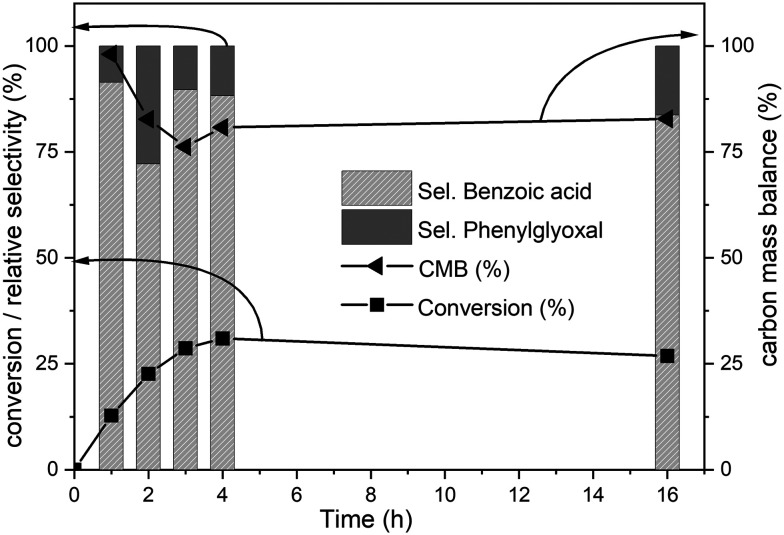
Time online data for the RICO of biphenyl. Biphenyl conversion (-■-), carbon mass balance (-◀-), the selectivity of benzoic acid (
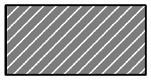
) and selectivity of phenylglyoxal (
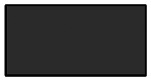
). The selectivity data reported here are relative selectivity between the two products. Reaction condition: biphenyl: 75 mg; NaIO_4_: 80 mg; RuCl_3_*x*H_2_O stock solution: 0.23 mL; 2 : 1 acetonitrile–deionised water solvent mixture: 30 mL; temperature: 22 °C and atmospheric pressure. Molar ratio of substrate : oxidant : catalyst = 1 : 2.67 : 0.03.

The time-on-line evolution of the products is presented in Fig. 3. Throughout the reaction, the selectivity of benzoic acid was between *ca.* 75–80% and the rest being phenyl glyoxal. Throughout the reaction the carbon balance was *ca.* 80% considering only benzoic acid and phenyl glyoxal for the calculation. After 4 h, 30% of biphenyl is converted to benzoic acid with 75% selectivity. The reaction reaches a plateau after 4 h and this could because of the consumption of the catalyst and/or oxidant. Both these products contain only one phenyl ring, and the other ring gets oxidized to CO_2_. The major product, benzoic acid, still contains the C–C bond, however this is a more reactive bond, compared to the original 5–5′ bond present in biphenyl. Benzoic acid can easily be decarboxylated to benzene, where the C–C bond is completely cleaved. The carbon balance for this reaction was *ca.* 80% because of the conversion of one phenyl ring to CO_2_. It is known that RICO oxidizes phenyl ring to CO_2_ in polyaromatic systems.^18,20^

**Fig. 4:**
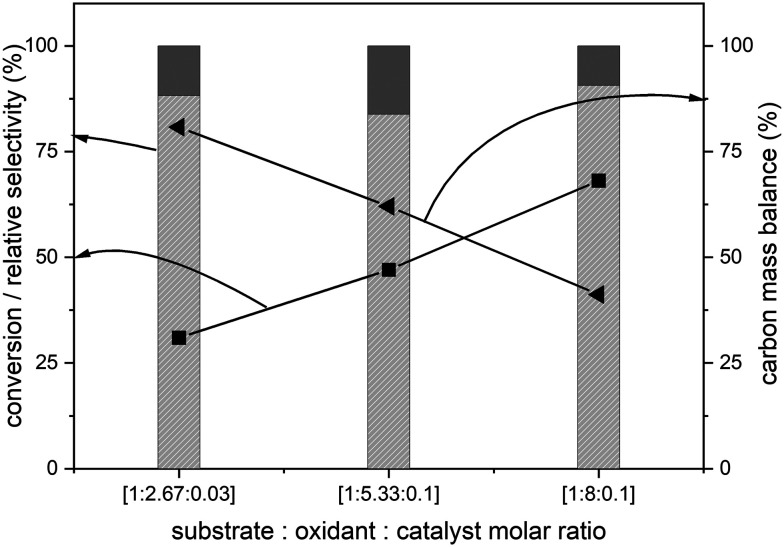
Effect of the amount of oxidant during the RICO of biphenyl. Biphenyl conversion (-■-), carbon mass balance (-◀-), the selectivity of benzoic acid (
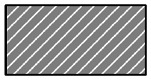
) and selectivity of phenylglyoxal (
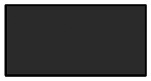
). The selectivity data reported here are relative selectivity between the two products. Reaction condition: biphenyl: 75 mg; NaIO_4_: 80 mg; RuCl_3_*x*H_2_O stock solution: 0.23 mL; 2 : 1 acetonitrile–deionised water solvent mixture: 30 mL; temperature: 22 °C and atmospheric pressure.

The amounts of catalyst and oxidant were varied to increase the conversion and the results are presented in Fig. 4. Increase in the amount of oxidant resulted in a substantial improvement in the biphenyl conversion from 30% to 70%. However, this increase was accompanied by a huge decrease in the carbon mass balance to less than 50% (Fig. 4). This reduction is due to a combination of two factors – oxidation of one of the phenyl rings to CO_2_ and the increase in the formation of unknown products confirmed by the increase in the peak areas of two unknown products. Further GC-MS studies suggested that these are cinnamaldehyde and *trans*-cinnamic acid (ESI,† Fig. S6). Detailed isolation and purification studies are needed to confirm these products. These initial studies on the ruthenium catalysed oxidation of biphenyl have resulted in products with a more reactive inter-unit C–C linkage confirming the activation of the 5–5′ linkage between the two phenyl rings. This is the first ever report on the catalytic oxidative activation of this C–C bond in biphenyl. Computational methods were employed to understand the mechanism of this reaction.

### Computational studies

**Fig. 5:**
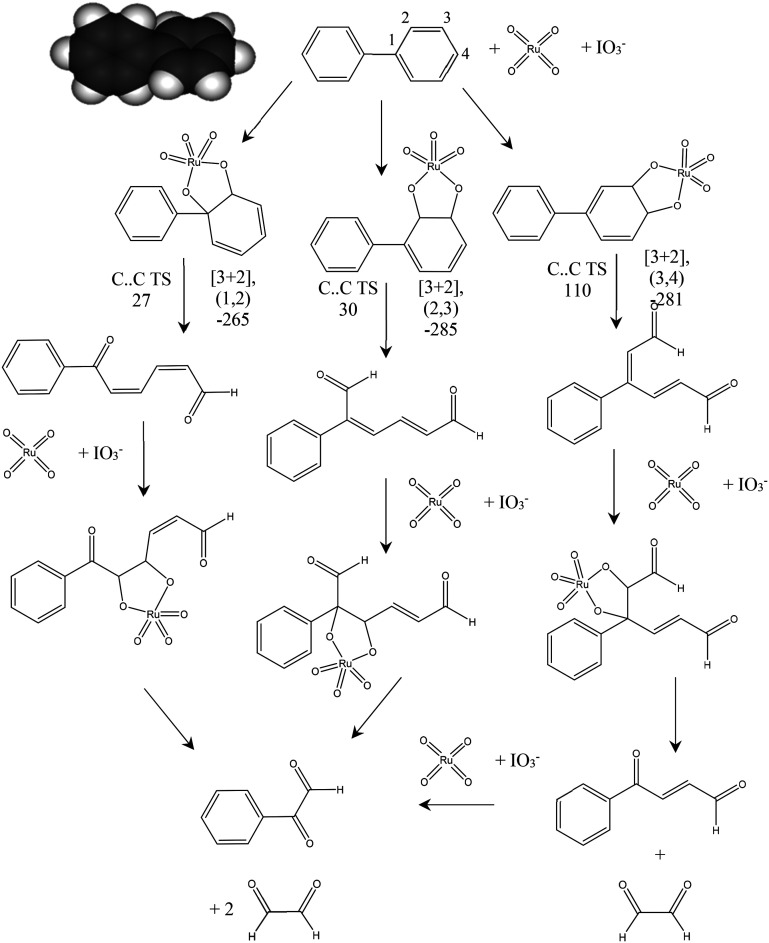
Proposed mechanism for the oxidation of biphenyl using ruthenium tetroxide. [3 + 2] formation energies are quoted for the Ru^6+^ complex prior to oxidation with IO^3−^ to the Ru^8+^ oxidation state form of the complex shown. Energies for C–C bond cleavage transition states (TS) are quoted for the forming Ru^8+^ state. Graphical inset shows the optimised geometry of biphenyl. Atom colours C: grey, H: white. All energies quoted in kJ mol^−1^.

Fig. 5 shows the proposed reaction pathways by which RICO applied to biphenyl can lead to the observed phenylglyoxal product. The adduct formation energy for the addition of RuO_4_ to biphenyl was calculated for a number of positions including co-ordination of the bridging C–C bond (1,1′), a [3 + 4] adduct by co-ordination to carbons (2,2′) and the formation of [3 + 2] adducts at the three unique C–C bonds in one aromatic ring. Formation of the [3 + 2] adduct using neighbouring C atoms within the same aromatic ring was found to be consistently 100 kJ mol^−1^ more favourable than the adducts formed with one carbon from each ring. Suggesting that oxidation of the biphenyl will be initiated at one of the aromatic rings rather than immediately cleaving the bridging C–C bond.

In our previous work on the oxidation of polyaromatic hydrocarbons it was found that the transition state for C–C cleavage within a [3 + 2] adduct was significantly lower if it is assumed that the Ru centre is first oxidised to Ru^8+^ by IO_3_^−^ than if C–C bond cleavage takes place from the Ru^6+^ state of the initial [3 + 2] adduct.^20^ This was also the case here, so Fig. 5 gives the barriers to C–C cleavage from the structures shown in the mechanism. The (1,2) and (2,3) positions show a similar barrier to reaction while the calculated barrier to cleavage for the (3,4) adduct is considerably higher. In all three cases the C–C bond scission leads to isolated alkene bonds and calculation of the formation energy of a new [3 + 2] adduct at these sites is found to be more than 100 kJ mol^−1^ more negative than was found for the formation of the [3 + 2] adduct on the aromatic ring system of biphenyl. This implies that, once an aromatic ring is opened RuO_4_ will rapidly catalyse the further oxidation of the side group formed. Fig. 5 also demonstrates that this process will lead to phenylglyoxal irrespective of the initial position of the reaction of the aromatic ring.

From these results we suggest that phenylglyoxal is the initial product of RICO for biphenyl, with benzoic acid being formed as a further oxidation product either from the RICO chemistry itself or as a result of our experimental work up for product analysis. The oxaldehyde (C2 aldehyde) proposed in this mechanism was not detected experimentally.

### Oxidative depolymerisation of model hexamer

Most of the literature on the development of catalytic systems either use simple monomeric or dimeric model compounds or lignin itself.^23^ Most model compounds do not adequately represent the complexity present in real lignins whereas, real lignins are too complex for developmental studies and analyses. To bridge this wide gap, Forsythe *et al.* reported the synthesis of a model oligomer (called as hexamer) containing β-O-4′, 5–5′ and β-5′ linkages (Fig. 5).^24^ This is an ideal model compound to study the fate of other inter-unit linkages during the oxidative cleavage of 5–5′ linkage. RICO of this hexamer was conducted like biphenyl oxidation, but without the acid-washing step.

### GPC analyses

The reaction was performed with the substrate : oxidant : metal molar ratio of 1 : 8 : 0.1 for 0.5 h, 2 h and 16 h at 22 °C. The dry solid products, after vacuum drying, were dissolved in THF to determine the molecular weight of the product(s) using gel permeation chromatography (GPC). The Mw of the starting material (hexamer) is 1047.19 g mol^−1^, and the GPC data presented in Fig. 7 clearly shows the complete depolymerisation of the hexamer after 2 h.

### NMR spectroscopic analyses

The next step was to quantitatively analyse the conversion of individual inter-unit linkages in the hexamer during the RICO reaction. NMR spectroscopy has been widely used as a qualitative and quantitative methodology for the structural characterisation of lignin.^25–28^^1^H NMR spectra of the product mixtures sampled after different times are presented in Fig. 8 and they clearly indicate, after 1 h, most of the inter-unit linkages have got converted. Two peaks at *δ* 4.2 ppm and *δ* 4.9 ppm, corresponding to the α and β Hs of the β-O-4 linkages, have disappeared after 60 min (Fig. 8). This suggests the conversion of the β-O-4 linkage in the hexamer. Similarly, the disappearance of the peak at *δ* 5.1 ppm suggests the conversion of the β-5 inter-unit linkage. Quantitative studies were done using a sealed glass insert with an accurate amount of tetramethylsilane (TMS) in CDCl_3_ as the external standard. Response factors, calculated using calibration plots, were used for these quantification analyses. They reveal (Fig. 9) 95% of the β-O-4 and 80% of the β-5 linkages are converted after 2 h of the reaction and this complements the GPC data presented in Fig. 7. However, ^1^H NMR spectroscopy could not be used to study the fate of the 5–5′ C–C linkage during the reaction, hence, ^13^C NMR spectroscopy was employed for this. The peak at *δ* 124 ppm correspond to the Cs in the 5–5′ C–C linkage present in the hexamer. ^13^C NMR spectra of the reaction mixtures sampled at different time (Fig. 10) shows that this peak disappears after 1 h, which suggests that there is a complete conversion of this 5–5′ C–C linkage after 1 h. However, this is not accurate because of the lack of sensitivity of the ^13^C nuclei at low concentration for NMR quantification. Hence, advanced 2D heteronuclear multiple bond correlation (HMBC) methodology was used for the calibration and the quantification of the 5–5′ bond cleavage in the hexamer during the reaction. The results (Fig. 11) still indicates that rate of 5–5′ conversion is equal or higher than that of β-5 conversion.

Phosphitylation of the free hydroxyl groups using chlorophosphite reagents, followed by ^31^P NMR spectroscopic characterisation, is commonly used for the characterisation of lignins and biofuels precursors.^25–27^^31^P NMR spectroscopy of the derivatized compound is also an useful tool for the quantification of functional groups such as carboxylic acids, phenols and aliphatic hydroxyl groups in complex organic molecules such as lignins.^27^ Typically, either 2-chloro-1,3,2-dioxaphospholane (DP) or 2-chloro-4,4,5,5-tetramethyl-1,3,2-dioxaphospholane (TMDP) is used as the phosphitylation reagent (structures of DP and TMDP are presented in Fig. 12). A list of ^31^P NMR chemical shifts of typical hydroxyl functional groups, after derivatization, present in lignin is presented in Table S4 (ESI†). DP is used to distinguish primary (γ-OH) from the secondary (α-OH) alcohols closer to the β-O-4 linkage in the hexamer. Fig. S7 and S8 (ESI†) gives the ^31^P NMR spectra of the hexamer, after derivatization by DP (Fig. S7†) and TMDP (Fig. S8†) show different peaks corresponding to γ-OH and secondary α-OH groups. DP can also be used to distinguish between *erythro*- and *threo*-conformations of β-O-4 structures. On the other hand, TMDP is effective in quantifying the guaiacyl (G-units) and syringyl (S-units) phenolic units in lignin. In this work, DP was used as the phosphitylation agent to quantitatively analyse the conversion of the 5–5′ linkages in the hexamer. For ^31^P NMR spectroscopic measurements, pyridine and CDCl_3_ (1.6 : 1, v/v) was used as solvent. Pyridine is added to neutralize the HCl formed during the phosphitylation reaction. For quantitative measurements, (*N*-hydroxyphthalimide) was used as the internal standard.^26^ A list of different internal standards and their ^31^P NMR chemical shifts are presented in the ESI† (Table S5). For all the NMR spectroscopic measurements, chromium(iii) acetylacetonate (Cr(acac)_3_) was added as the relaxation agent to lower the spin–lattice relaxation time of the ^31^P nuclei thereby shortening the measurements time significantly.^25^

**Fig. 6:**
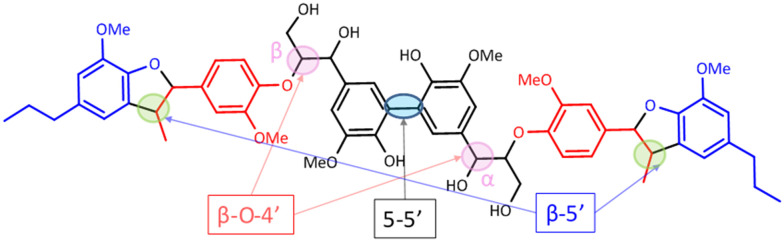
Structure of the hexamer used where the inter-unit linkages are marked.

**Scheme 2:**
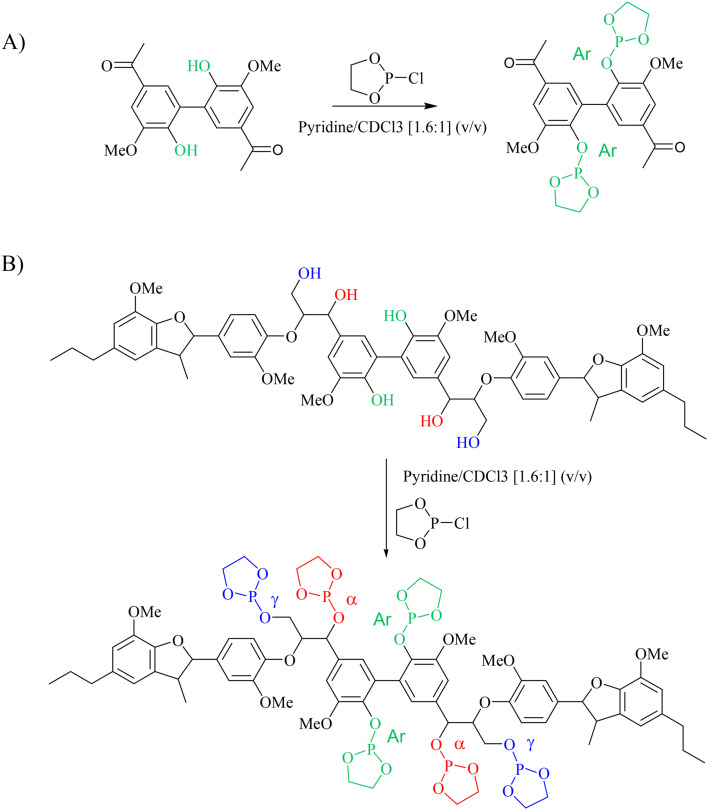
Phosphitylation of the 5–5′ dimer (A) and hexamer (B) using DP.

The starting material (hexamer) (Fig. 6), a standard dimer molecule containing only 5–5′ linkage (Scheme 2), internal standard and the products after RICO reaction were derivatized (phosphitylation) with DP and their ^31^P NMR spectra were recorded (Fig. 13). The ^31^P shift of the internal standard (*N*-hydroxyphthalimide) was observed at 135.5 ppm, whereas the peak between 130 and 130.7 ppm correspond to the aromatic OH groups which represents the 5–5′ linkage, which is in line with the reported values.^25–27^

**Fig. 7:**
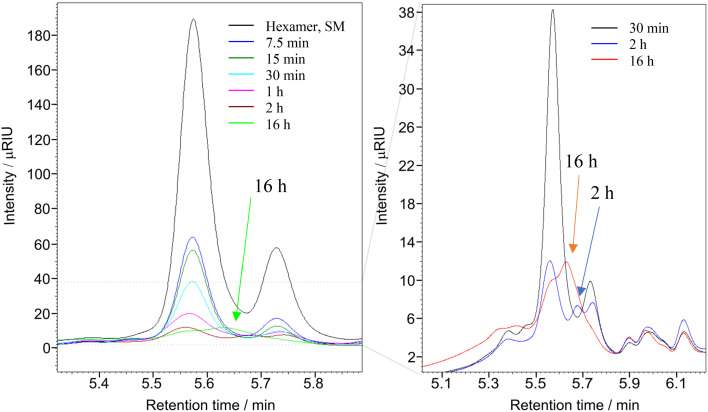
Overlap GPC chromatogram of the reaction products of hexamer in THF at different reaction times after RICO reaction. Left: Reaction from 7.5 min to 16 hours of reaction, including the starting material (black line). Right: Zoom of the chromatogram for reaction products obtained after 30 min, 2 h and 16 hours. 171.7 mg, 0.196 mmol of hexamer, 280 mg NaIO_4_ and 0.23 mL of RuCl_3_·*x*H_2_O stock solution. 30 mL of reaction solvent mixture of 2 : 1 acetonitrile–deionised water, at 22 °C, 1 atm.

^31^P NMR spectroscopic data of the derivatized hexamer allows us to quantify the ratio of diastereomeric isomers. In our sample, we found a high percentage of *erythro* and a small percentage of *threo* diastereoisomer.^24^ GPC data (Fig. 7) shows that both diastereomers depolymerized during the RICO reaction. Detailed list of peaks, integration values used for this quantitative analysis is give in the ESI† (Table S6). The reaction mixtures, sampled after 15 and 30 min of the RICO reaction were phosphitylated using DP and their ^31^P NMR spectra were recorded (Fig. 14). The peak at 130.5 ppm (corresponding to the 5–5′ linkage), disappeared after 30 min, proving the complete conversion of the 5–5′ C–C interunit linkage after 30 min of the reaction. This data complements the conclusion derived from the quantitative studies using HMBC and the GPC.

**Fig. 8:**
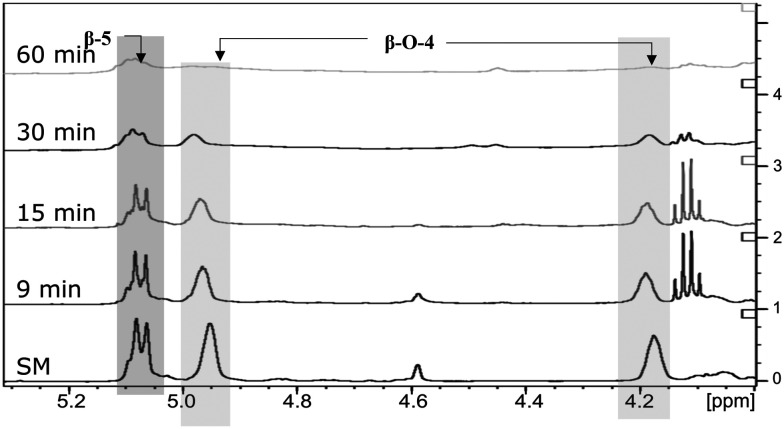
Comparison of ^1^H NMR spectra: from bottom to top – starting material (SM), products obtained after 9 min, 15 min, 30 min and 1 h of RICO reaction.

**Fig. 9:**
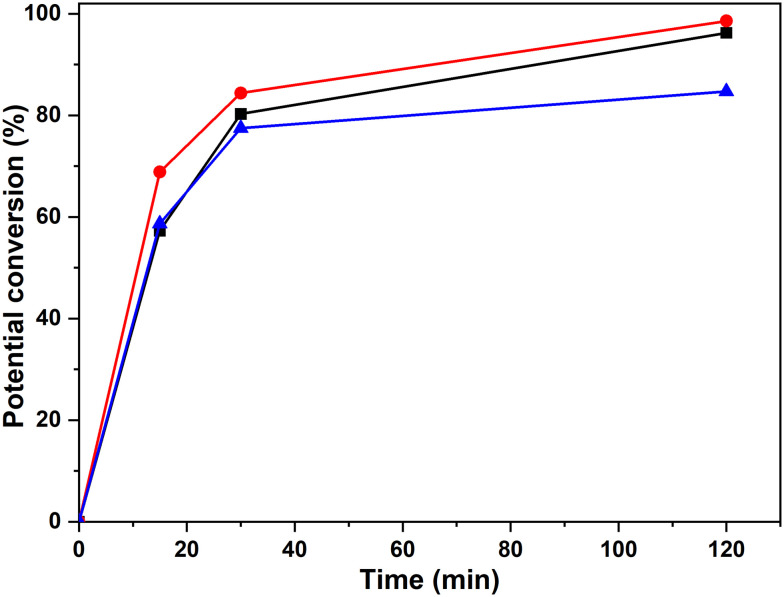
Potential percentage of inter-unit bond conversion corresponding to the bonds β-O-4′(β) (-■-), β-O-4′(α) (-●-) and β-5′ (-▲-), obtained by ^1^H-NMR spectra with the integration of the areas at *δ*_H_ of 4.19 ppm, 4.98 ppm and 5.08 ppm, respectively.

**Fig. 10:**
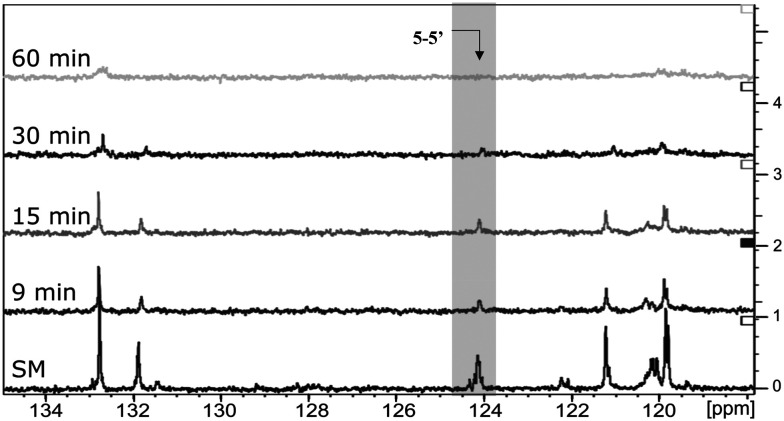
Comparison of ^13^C NMR spectra: from the bottom to the top – starting material (SM), products obtained after 9 min, 15 min, 30 min and 1 h of RICO reaction.

**Fig. 11:**
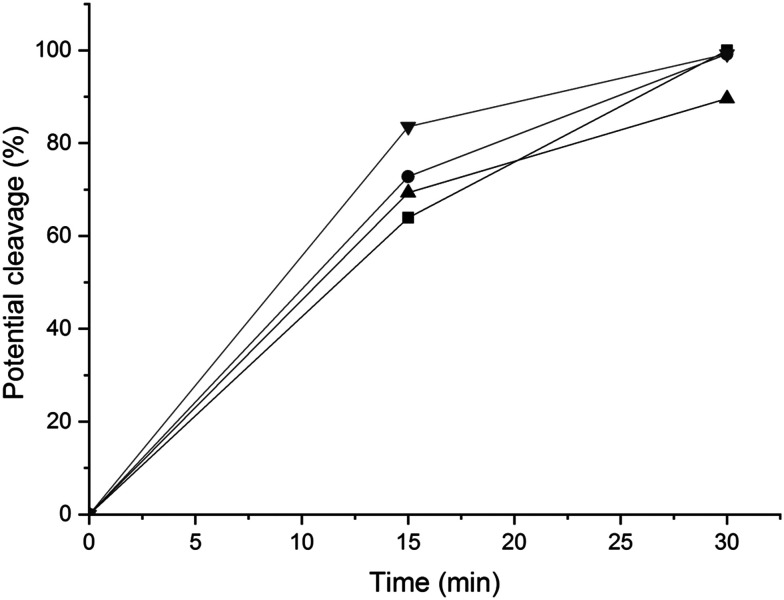
Potential conversion of the β-O-4′(β) (-■-), β-O-4′(α) (-●-) and β-5′ (-▲-) and 5–5′ (-▼-) environments according to the HMBC spectra with the integration of the signals at (4.19, 146.77 ppm), (4.97, 108.42 ppm), (5.08, 110.29 ppm) and (6.89, 124.09 ppm) respectively.

**Fig. 12:**
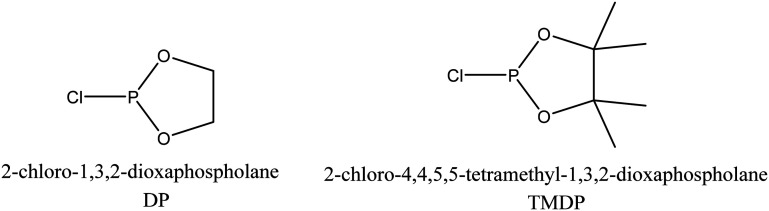
Structure of phosphite reagents.

**Fig. 13:**
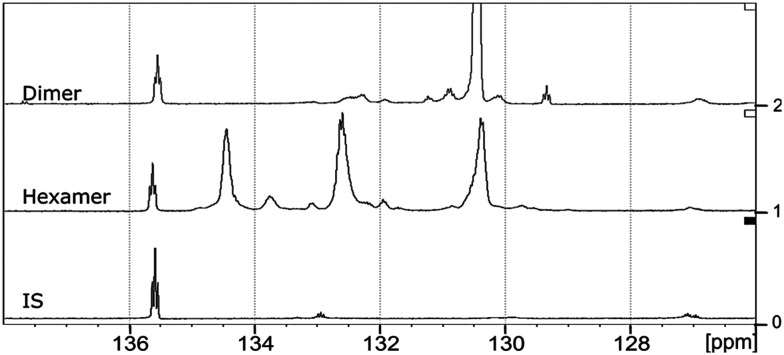
^31^P NMR spectra of the dimer, hexamer and *N*-hydroxyphthalimide (IS) after derivatization using DP.

**Fig. 14:**
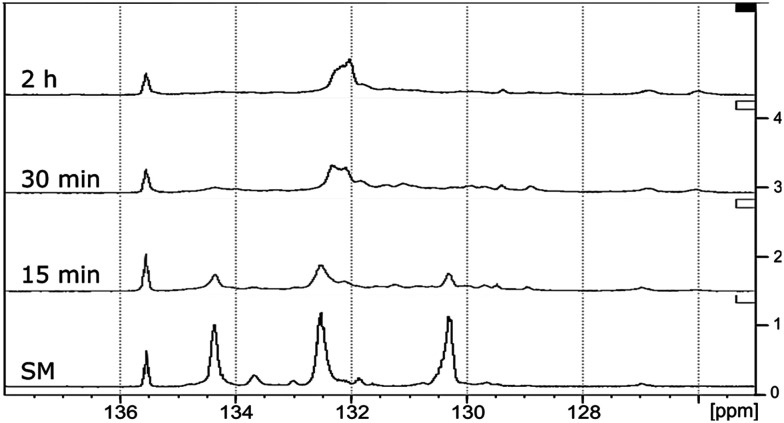
^31^P NMR spectra of the starting material of the hexamer (SM) followed by the dry oxidation products after 15 min, 30 min and 2 h of RICO reaction.

**Fig. 15:**
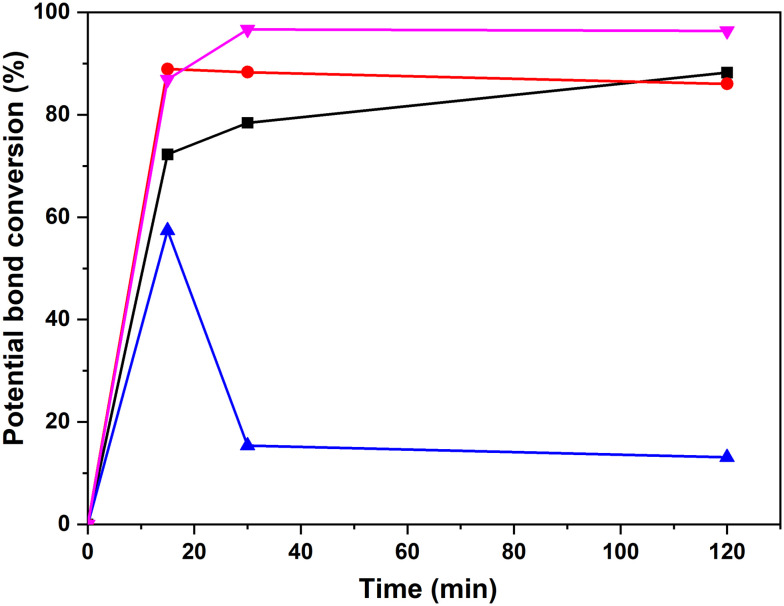
Time online studies of the potential percentage conversion of the bonds β-O-4′ α-OH *erythro* (-■-), β-O-4′ α-OH *threo* (-●-), β-O-4′ γ-OH (-▲-) and the 5–5′ Ar–OH (-▼-) obtained by ^31^P-NMR spectroscopy.

Quantitative analyses of the conversion of the 5–5′ and β-O-4 linkages in the hexamer were done using the internal standard and peak normalisation methodology. Fig. 15 shows the time online conversion of the 5–5′ and β-O-4 inter-unit linkages. After 30 min of the reaction, *ca.* 88% of the 5–5′ linkages are converted, which approximately agrees with the values obtained from the HMBC analyses. These data clearly demonstrate the effectiveness of RICO methodology to convert the most recalcitrant 5–5′ interunit linkage in lignins. Recently Zhu *et al.* reported the activation of 2–2′ C–C linkage in biphenols using a catalytic hydrogenolysis methodology.^29^ To the best of our knowledge, this is one of the most effective catalytic methodologies to activate or convert the most recalcitrant C–C interunit linkage in lignins. Consequently, this methodology can be used for the depolymerisation of lignins where C–C linkages dominate, for example technical lignins.

### Products identification

**Fig. 16:**
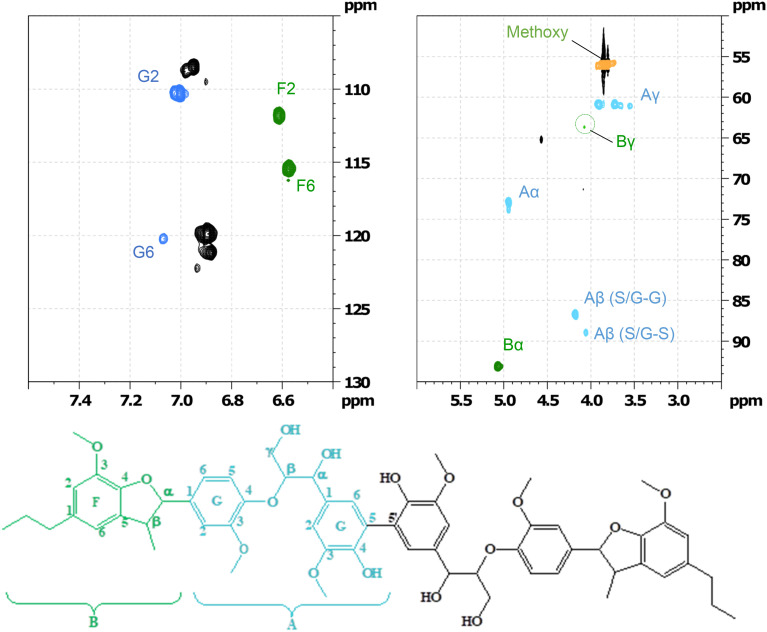
Partial HSQC spectrum of the starting material and its corresponding assignment corresponding to labelled on the hexamer structure.

GC-MS as well as HSQC-NMR spectroscopic techniques were employed to identify some of the products after the RICO of the hexamer. Initially, the structure of the starting material (hexamer) was characterised by HSQC NMR spectroscopic technique (Fig. 16). Table S7† lists different functional groups corresponding to different inter-unit linkages present in the hexamer and their *δ*_C_/*δ*_H_ assignment in the HSQC spectra.^30,31^

**Fig. 17:**
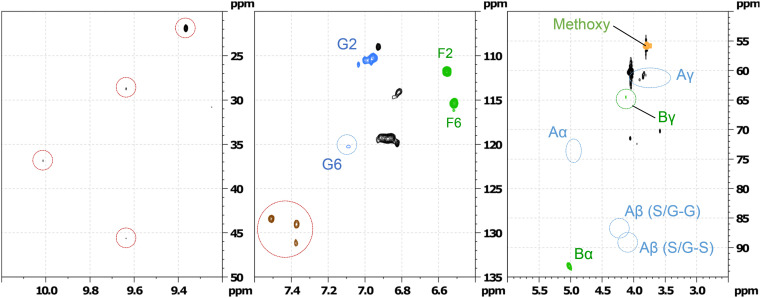
Partial HSQC spectrum of the fraction 1 separated by column chromatography of the oxidation of hexamer after 1 h of RICO reaction.

Column chromatography was used to separate the products from the products mixture after the reaction. The different fractions were dried in vacuum and re-dissolved in ethyl acetate for GC-MS analysis. Table S8 (ESI†) lists all the potential compounds identified by GC-MS from all these fractions. This list is indicative, not confirmative, because while some of these products are likely, others are unlikely from the reaction. Hence, HSQC NMR spectroscopy method was employed to identify major products. HSQC of the 1st fraction (F1) (Fig. 17) shows that all the β-O-4′ sub-structures (indicated by A in Fig. 16) are not present in this fraction, however, new aromatic region were found at *δ*_H_/*δ*_C_ 7.5/130, which could be assigned to C_2,6_–H_2,6_ in *p*-hydroxybenzoate (PB) substructures along with possible aldehyde functionalities.^32,33^ This suggests the oxidative conversion of the β-O-4 linkage present in the hexamer. However, the HSQC of the second fraction (F2) (ESI† Fig. S13) suggests the presence of the β-O-4 linkage along with some aldehydes. This suggests that the β-O-4 linkage is not completely converted and confirms the results obtained from the quantitative studies (Fig. 15).

The HSQC data (Fig. 17) of the 1st fraction (F1) further suggests that the β-5′ inter-unit linkage in the phenylcoumaran substructure (indicated by B in Fig. 16) is still present to some extent, and probably one of the G aromatic rings in A is also present in the products mixture because of the presence of F6 and G2 confirmed by the presence of the peaks at *δ*_H_/*δ*_C_ 6.5/115 and 7.0/110 ppm, respectively. It is not surprising that this β-5′ inter-unit linkage survived this RICO reaction, because it is one of the robust inter-unit linkages present in lignin. HSQC analysis of the second fraction (F2) suggests the presence of small amounts of compounds containing 5–5′ linkage as well (2-phenyl-4,5-methylenedioxybenzaldehyde, 2-(2,6-dimethoxyphenyl)-5,6-dimethoxy-4*H*-chromen-4-one). Another major compound identified by both GC-MS as well HSQC NMR spectroscopy is 2-methoxy-4-(7-methoxy-3-methyl-5-propyl-2,3-dihydrobenzofuran-2-yl(phenol) (β-5 compound). Fig. S9–S12 (ESI†) shows the HSQC spectra of the first fraction (F1) and the structure of the β-5 compound. This compound is present in both the chromatographic fractions (F1 & F2) and this is not surprising because it is one of the intermediates during the synthesis of the hexamer model compound.^24^ More in-depth analysis and studies are necessary to identify the reaction mechanism and the pathways during the ruthenium ion catalysed oxidative depolymerisation of the hexamer.

## Conclusion

We report that the RICO reaction is an effective catalytic system for the activation and conversion of the most recalcitrant C–C inter-unit linkage (5–5′) present in lignin. Initial studies using biphenyl as the model compound for the 5–5′ linkage resulted in the formation of benzoic acid (major product) and phenyl glyoxal (minor product) *via* the oxidation of one of the phenyl rings. Though, the original C–C inter phenyl unit linkage, is present in benzoic acid, it is significantly easier to break this bond *via* the decarboxylation or other methodologies. By optimizing the reaction conditions, *ca.* 65% biphenyl conversion was achieved accompanied by reduced benzoic acid selectivity and carbon balance. This is due to the formation of unidentified products and CO_2_. However, this data set suggests that the conversion/selectivity can be tuned by optimizing the reaction conditions such as solvent system and catalyst/substrate/oxidant ratio. DFT studies with the hybrid B3LYP functionals of this system revealed that RuO_4_ forms a [3 + 2] adduct with one of the aromatic C–C bonds resulting in the opening of the phenyl ring.

Since RICO is an effective catalyst system to activate the most recalcitrant C–C linkage in lignin, we employed this for the depolymerization of a lignin model hexamer containing β-O-4′, β-5′ and 5–5′ linkages. GPC and mononuclear NMR spectroscopic analyses revealed that the hexamer is completely depolymerized after 2 h. Quantitative analyses of the conversion of β-O-4′ and β-5′ linkages were done using ^1^H NMR spectroscopy using sealed TMS insert (internal standard). After 2 h of the reaction *ca.* 80% of both linkages are converted. HMBC 2D-NMR spectroscopy and ^31^P-NMR spectroscopy (after P derivatization) were used for the quantitative analyses of the conversion of the 5–5′ linkage. These analyses revealed that after 30 min of the reaction, >95% of the 5–5′ linkages are converted in the hexamer. To the best of our knowledge, this is the most active system ever reported for the conversion of the recalcitrant 5–5′ linkage to smaller units. Preliminary products identification was carried out using GC-MS and 2D-NMR spectroscopic methods.

## Conflicts of interest

There are no conflicts to declare.

## Supplementary Material

CY-013-D3CY00076A-s001

## References

[cit1] Zakzeski J., Bruijnincx P. C. A., Jongerius A. L., Weckhuysen B. M. (2010). Chem. Rev..

[cit2] Li C., Zhao X., Wang A., Huber G. W., Zhang T. (2015). Chem. Rev..

[cit3] Kubicek C. P. , Fungi and Lignocellulosic Biomass, John Wiley & Sons, Inc., 2012, pp. 1–28

[cit4] Luo H. and Abu-Omar M. M., in Encyclopedia of Sustainable Technologies, ed. M. A. Abraham, Elsevier, Oxford, 2017, pp. 573–585

[cit5] Liu X., Bouxin F. P., Fan J., Budarin V. L., Hu C., Clark J. H. (2020). ChemSusChem.

[cit6] Zhang C. and Wang F., in Advances in Inorganic Chemistry, ed. P. C. Ford and R. van Eldik, Academic Press, 2021, vol. 77, pp. 175–218

[cit7] Jing Y., Dong L., Guo Y., Liu X., Wang Y. (2020). ChemSusChem.

[cit8] Ročnik T., Likozar B., Jasiukaitytė-Grojzdek E., Grilc M. (2022). Chem. Eng. J..

[cit9] Parthasarathi R., Romero R. A., Redondo A., Gnanakaran S. (2011). J. Phys. Chem. Lett..

[cit10] Guadix Montero S., Meenakshisundaram S. (2018). Top. Catal..

[cit11] Rinaldi R., Jastrzebski R., Clough M. T., Ralph J., Kennema M., Bruijnincx P. C. A., Weckhuysen B. M. (2016). Angew. Chem., Int. Ed..

[cit12] Djerassi C., Engle R. R. (1953). J. Am. Chem. Soc..

[cit13] Plietker B. (2005). Synthesis.

[cit14] Mills A., Holland C. (1997). J. Chem. Res., Synop..

[cit15] Carlsen P. H. J., Katsuki T., Martin V. S., Sharpless K. B. (1981). J. Org. Chem..

[cit16] Piccialli V. (2014). Molecules.

[cit17] Méndez A., Bermejo J., Santamaría R., Blanco C. G., Menéndez R. (2000). Energy Fuels.

[cit18] Nowicka E., Meenakshisundaram S., Jenkins R. L., Knight D. W., Willock D. J., Hutchings G. J., Francisco M., Taylor S. H. (2015). Chem. – Eur. J..

[cit19] Frunzke J., Loschen C., Frenking G. (2004). J. Am. Chem. Soc..

[cit20] Nowicka E., Hickey N. W., Meenakshisundaram S., Jenkins R. L., Knight D. W., Willock D. J., Hutchings G. J., Francisco M., Taylor S. H. (2018). Chem. – Eur. J..

[cit21] Takahashi N., Hashimoto M., Tsukagoshi K. (2013). Anal. Sci..

[cit22] Frisch M. J. , Trucks G. W., Schlegel H. B., Scuseria G. E., Robb M. A., Cheeseman J. R., Scalmani G., Barone V., Petersson G. A., Nakatsuji H., Li X., Caricato M., Marenich A., Bloino J., Janesko B. G., Gomperts R., Mennucci B., Hratchian H. P., Ortiz J. V., Izmaylov A. F., Sonnenberg J. L., Williams-Young D., Ding F., Lipparini F., Egidi F., Goings J., Peng B., Petrone A., Henderson T., Ranasinghe D., Zakrzewski V. G., Gao J., Rega N., Zheng G., Liang W., Hada M., Ehara M., Toyota K., Fukuda R., Hasegawa J., Ishida M., Nakajima T., Honda Y., Kitao O., Nakai H., Vreven T., Throssell K., Montgomery, Jr. J. A., Peralta J. E., Ogliaro F., Bearpark M., Heyd J. J., Brothers E., Kudin K. N., Staroverov V. N., Keith T., Kobayashi R., Normand J., Raghavachari K., Rendell A., Burant J. C., Iyengar S. S., Tomasi J., Cossi M., Millam J. M., Klene M., Adamo C., Cammi R., Ochterski J. W., Martin R. L., Morokuma K., Farkas O., Foresman J. B. and Fox D. J., Gaussian 09, Revision A.02, Gaussian, Inc., Wallingford CT, 2016

[cit23] Lahive C. W., Kamer P. C. J., Lancefield C. S., Deuss P. J. (2020). ChemSusChem.

[cit24] Forsythe W. G., Garrett M. D., Hardacre C., Nieuwenhuyzen M., Sheldrake G. N. (2013). Green Chem..

[cit25] Pu Y., Cao S., Ragauskas A. J. (2011). Energy Environ. Sci..

[cit26] Zawadzki M., Ragauskas A. (2001). Holzforschung.

[cit27] Jiang Z.-H., Argyropoulos D. S., Granata A. (1995). Magn. Reson. Chem..

[cit28] Constant S., Wienk H. L. J., Frissen A. E., Peinder P. d., Boelens R., van Es D. S., Grisel R. J. H., Weckhuysen B. M., Huijgen W. J. J., Gosselink R. J. A., Bruijnincx P. C. A. (2016). Green Chem..

[cit29] Zhu J., Wang J., Dong G. (2019). Nat. Chem..

[cit30] Sette M., Lange H., Crestini C. (2013). Comput. Struct. Biotechnol. J..

[cit31] Yuan T.-Q., Sun S.-N., Xu F., Sun R.-C. (2011). J. Agric. Food Chem..

[cit32] Tan X., Jiang B., Yang Y., Min D., Jin Y. (2017). Holzforschung.

[cit33] Stewart J. J., Akiyama T., Chapple C., Ralph J., Mansfield S. D. (2009). Plant Physiol..

