# Supplementary material for: Ruthenium ion catalysed C–C bond activation in lignin model compounds – towards lignin depolymerisation
Source: Catal Sci Technol. 2023 Aug 25;13(20):5912–23. doi: 10.1039/d3cy00076a (PMC10577544; doi:10.1039/d3cy00076a)
Supplement: CY-013-D3CY00076A-s001 [file CY-013-D3CY00076A-s001.pdf]

Electronic Supplementary Material (ESI) for Catalysis Science & Technology.
This journal is © The Royal Society of Chemistry 2023

Supporting information

Ruthenium ion catalysed C-C bond conversion in lignin model compounds - Towards lignin depolymerisation.

Susana Guadix-Montero[a], Mala A. Sainna[a], W. Jiangpeiyun Jin[a], Jack Reynolds[a], Graham W. Forsythe[b], Gary N. Sheldrake[b], David Willock[a] and Meenakshisundaram Sankar[a]*

[a] Cardiff Catalysis Institute, School of Chemistry, Cardiff University, Cardiff CF10 3AT, United Kingdom.

[b] School of Chemistry and Chemical Engineering, David Keir Building, Stranmillis Road, Queens University Belfast, Belfast BT9 5AG, Northern Ireland, United Kingdom.

[*]Correspondence to Dr. M.Sankar Tel: +44 29 2087 5748, Fax: (+44) 2920-874-030, E-mail: sankar@cardiff.ac.uk

Methodology for the quantitative analyses using NMR Spectroscopy:

^{1}H NMR spectroscopy was applied for both qualitative and quantitative analyses. For quantitative analyses, the integrations of selected chemical shifts were measured and then normalised against the TMS peak. For example, the response factor for the hexamer was calculated by first dissolving three different known masses of the hexamer (10.8 mg, 20.5 mg, and 30.3 mg) in $CDCl_3$ (0.5 mL), using the same quantity of TMS sealed in a glass insert for all the samples. These samples were run through the NMR instrument (16 scans proton NMR method) and the relative areas for the desired proton environments were achieved from the integration of the resulting spectra. A plot of normalized peak area against the moles of the compound gave the response factor. The response factor for each proton environment of interest in the hexamer molecule was calculated using this methodology.

Figure S1 shows an example of one of the calibrations obtained, with the proton signal at δ 4.18 ppm. The response factor (gradient) was found to be 44867.

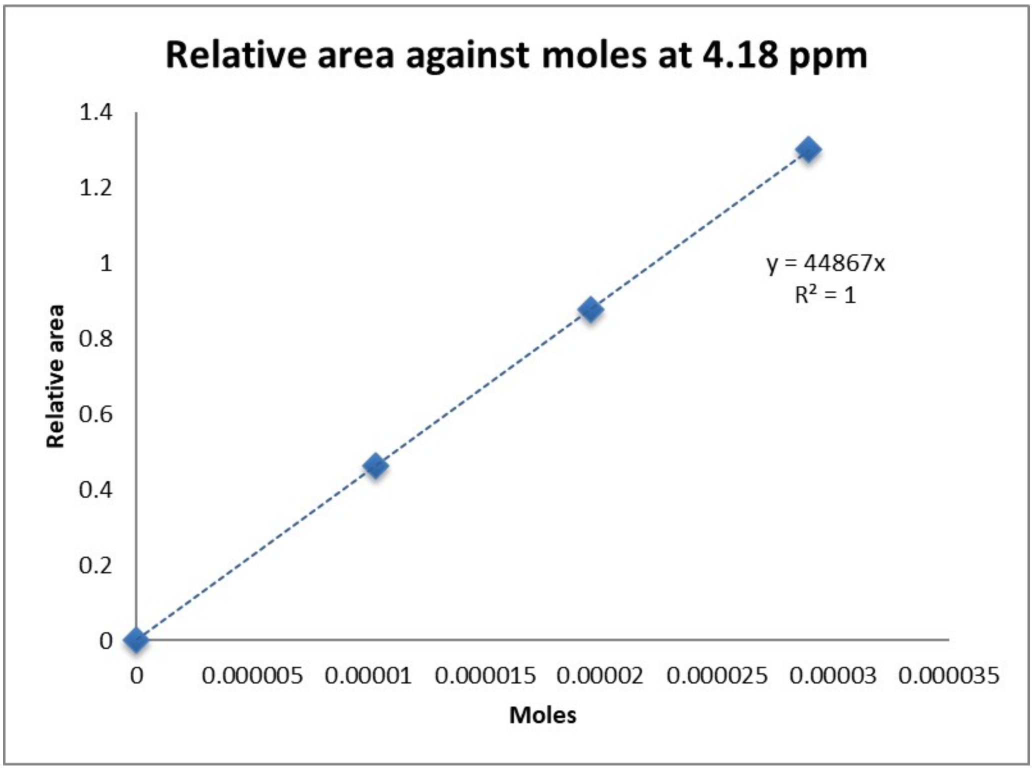

Figure: S1 Calibration of the hexamer using ^{1}H (peak at chemical shift (δ)= 4.18 ppm)

$$Response\ Factor\ (RF) = \frac{normalised\ area}{moles\ of\ hexamer}$$

After calculating the response factors for all the chemical shift (δ) of interest, the samples after RICO reaction, which contains unknown moles of the started material were analysed. Using the response factors, potential bond conversions for each inter-unit linkage were calculated. To estimate the potential conversion, the number of moles of the starting hexamer, the moles of the products and the moles of the product used in the NMR sample were used. Then, the actual number of moles of the hexamer within the sample was calculated using the response factors. This can then be scaled up to the total moles of hexamer in the total product mass. Finally, the potential bond conversion was calculated using the equation given below.

$$Potential\ bond\ conversion\ \ (\%) = \frac{(moles\ of\ hexamer\ at\ t = 0\ ) - (moles\ of\ hexamer\ after\ time\ t)}{moles\ of\ hexamer\ at\ t = 0}$$

Table S1 give the data after 30 min reaction.

Table S1 Estimation of potential conversion of different bonds using ¹H-NMR after 30 min RICO reaction of the hexamer.

RF	***Normalized Area***	**¹H Chemical shift δ (ppm)**	***Bond***	***Moles of NMR sample ($x10^{-6}$)***	***Moles of dry products ($x10^{-5}$)***	***Potential % conversion***
-	1	0	TMS	-	-	-
44867	0.2598	4.18	*β-O-4' (β)*	5.79	3.24	80.3
52254	0.2395	4.98	*β-O-4' (α)*	4.58	2.56	84.4
62206	0.4112	5.09	*β-5'*	6.61	3.70	77.5

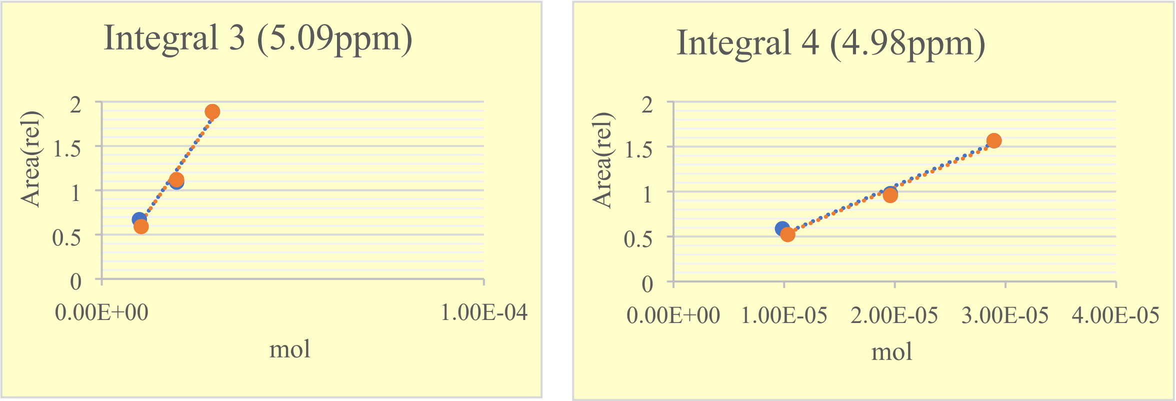

Figure S2 Calibration plots for the peaks at δ 5.09 ppm as well as δ 4.98 ppm.

2D NMR methods:

Heteronuclear multiple-bond correlation (2D-NMR) techniques such as HSQC and HMBC methods were used to quantitatively analyse bond conversion in hexamer. The methodology is similar to the above method, however, with an additional step of calibrate the axis and set the parameters to zero before integrating the TMS signal and normalising the peaks.

^{31}P NMR methods:

For quantitative analyses using ^{31}P NMR method, the samples were phosphorylated using different chlorophosphite reagents initially. Then, 40 mg of the hexamer or the dry material after the reaction was accurately weighed and dissolved in 400 µL of a solvent mixture of pyridine and $CDCl_3$ (1.6:1, v/v). 200 µL of the internal standard N-hydroxynophthalimide (11.4 mg mL^{-1}) in the solvent mixture and 50 µL of a stock solution of relaxation agent (11.4 mg mL^{-1} of $Cr(acac)_3$ in 5 mL of the solvent mixture) was added to the NMR tube. Typically in lignin analysis, a 25-s pulse delay is considered appropriate for quantitative ^{31}P-NMR. However, for the hexamer lignin model compound, it has been proved enough to use d1= 5 s, after confirmation of no change of signal for a range between 5 s and 15 s. The mixture was phosphorylated with 100 µL of 2-chloro-1,3,2-dioxaphospholane (DP) or its sterically hindered analogue 2-chloro-4,4,5,5-tetramethyl-1,3,2- dioxaphospholane (TMDP) depending on the OH groups to investigate. DP is better to distinguish between primary and secondary alcohols of the phenyl chains, carboxylic and guaiacyl phenolic hydroxyls, while TMDP is better to distinguish between guaiacyl and syringyl.

Using the known quantity of the internal standard, the amount of OH groups can be calculated using the ^{31}P signals (corresponding to the OH groups).

$$Moles\ of\ OH\ group = \frac{Area\ of\ the\ peak}{area\ of\ the\ IS} \times moles\ IS$$

Table S2 Quantitative analyses of sodium and ruthenium ions using ICP

Sample	**Na ions**	**Ru ions**
	Conc. [mg/l]	Conc. [mg/l]
Ethyl acetate phase	31	0.14
Aqueous phase	4546	88

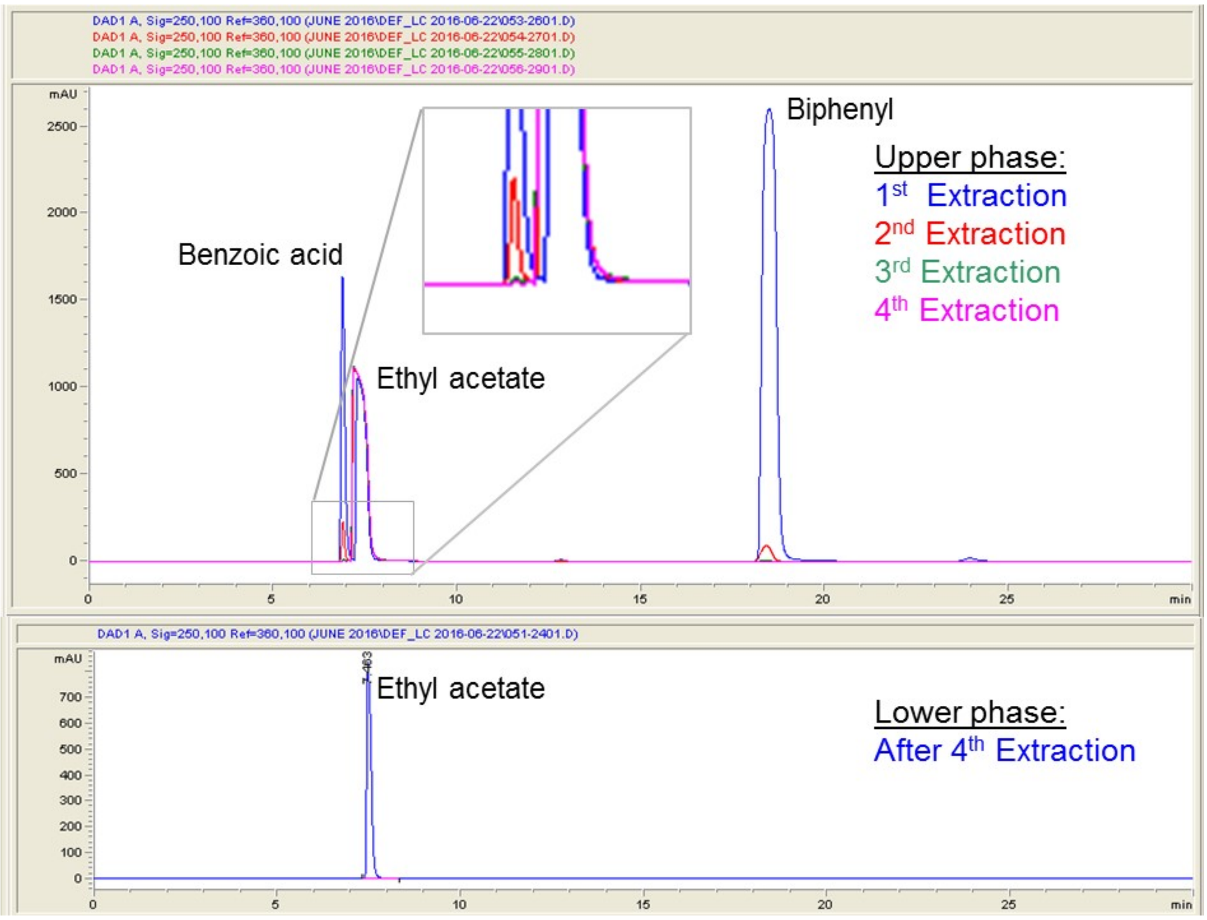

Figure S3: Control experiment for benzoic acid extraction. HPLC chromatograms of both ethyl acetate layer (upper phase) and aqueous layer (lower phase) after 4 extractions.

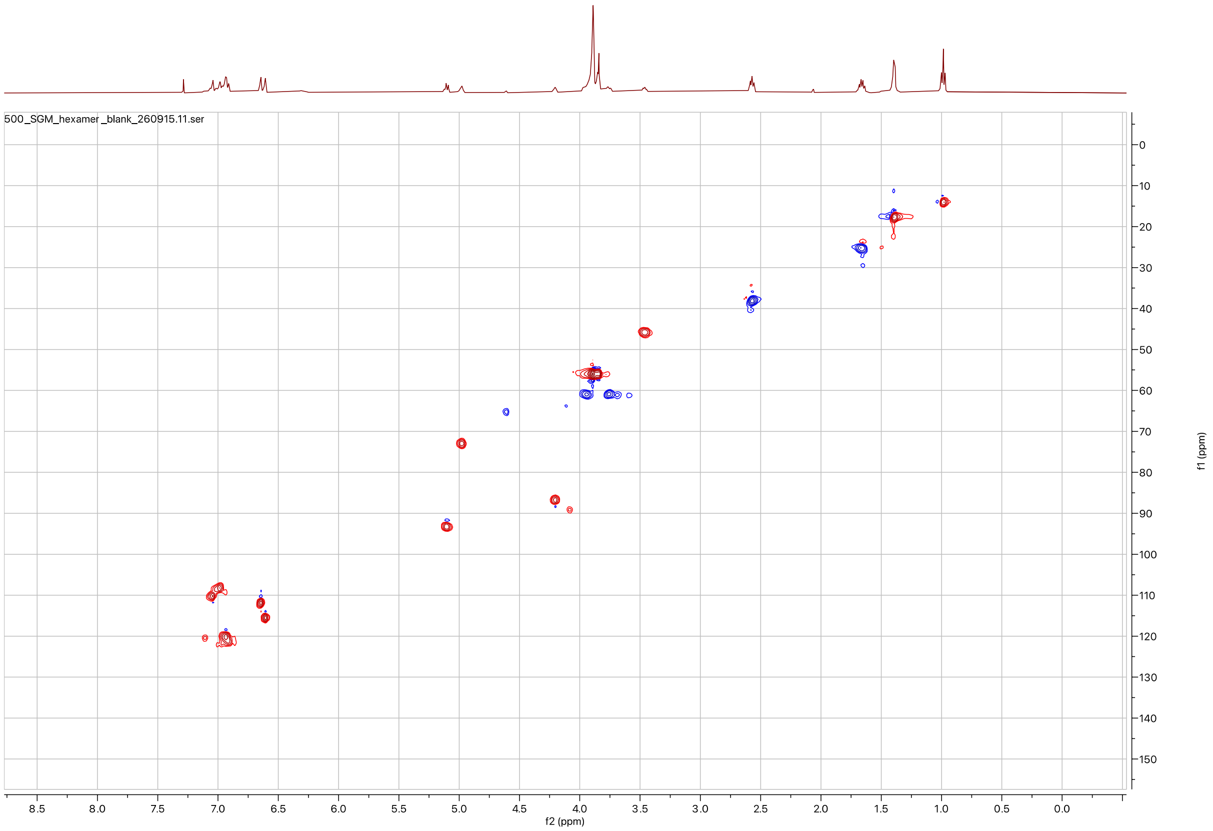

Figure S4: 2D NMR (HSQC) spectra of the blank reaction with oxidant alone without any catalyst. This reaction was carried out for 16h at 22 ^oC.

Table S3: Products identified by GC-MS in the biphenyl oxidation reaction mixture

#	*Retention time*	*Compound*	*Molecula r Formula*	*Structure*
1	3.01	Ethyl Propionate	$C_4H_8O_2$	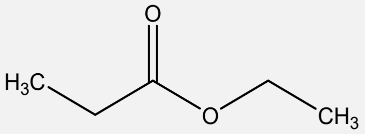
2	3.91	Toluene	C_7H_8	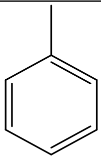
3	7.75	o-Xylene	C_8H_{10}	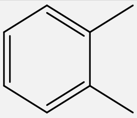
4	11.23	Benzaldehyde	C_7H_6O	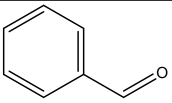
5	13.66	Phenylglyoxal	$C_8H_6O_2$	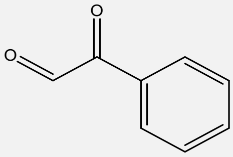
6	15.17	Benzoic acid	$C_7H_6O_2$	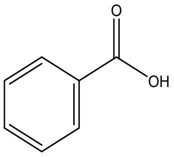
7	17.55	Cinnamaldehyde	C_9H_8O	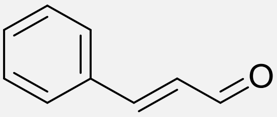
8	19.42	Biphenyl	$C_{12}H_{10}$	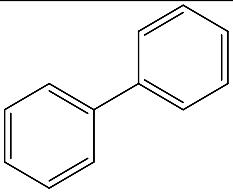

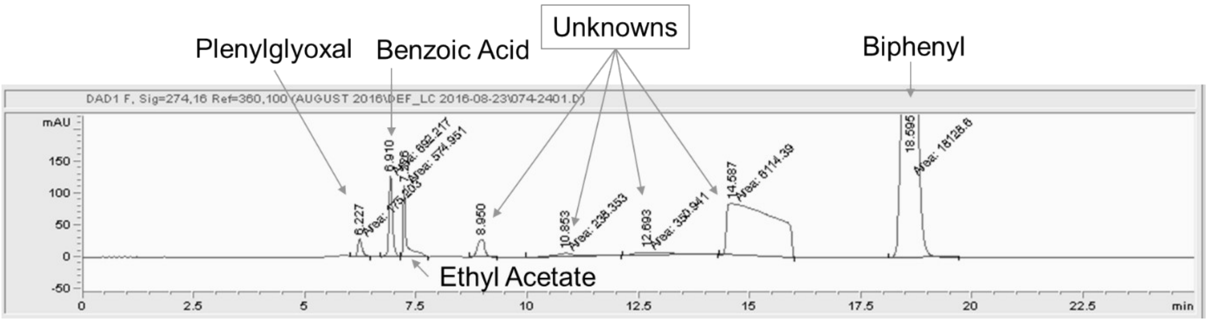

Figure S5 HPLC chromatogram of the reaction product after RICO reaction of Biphenyl.

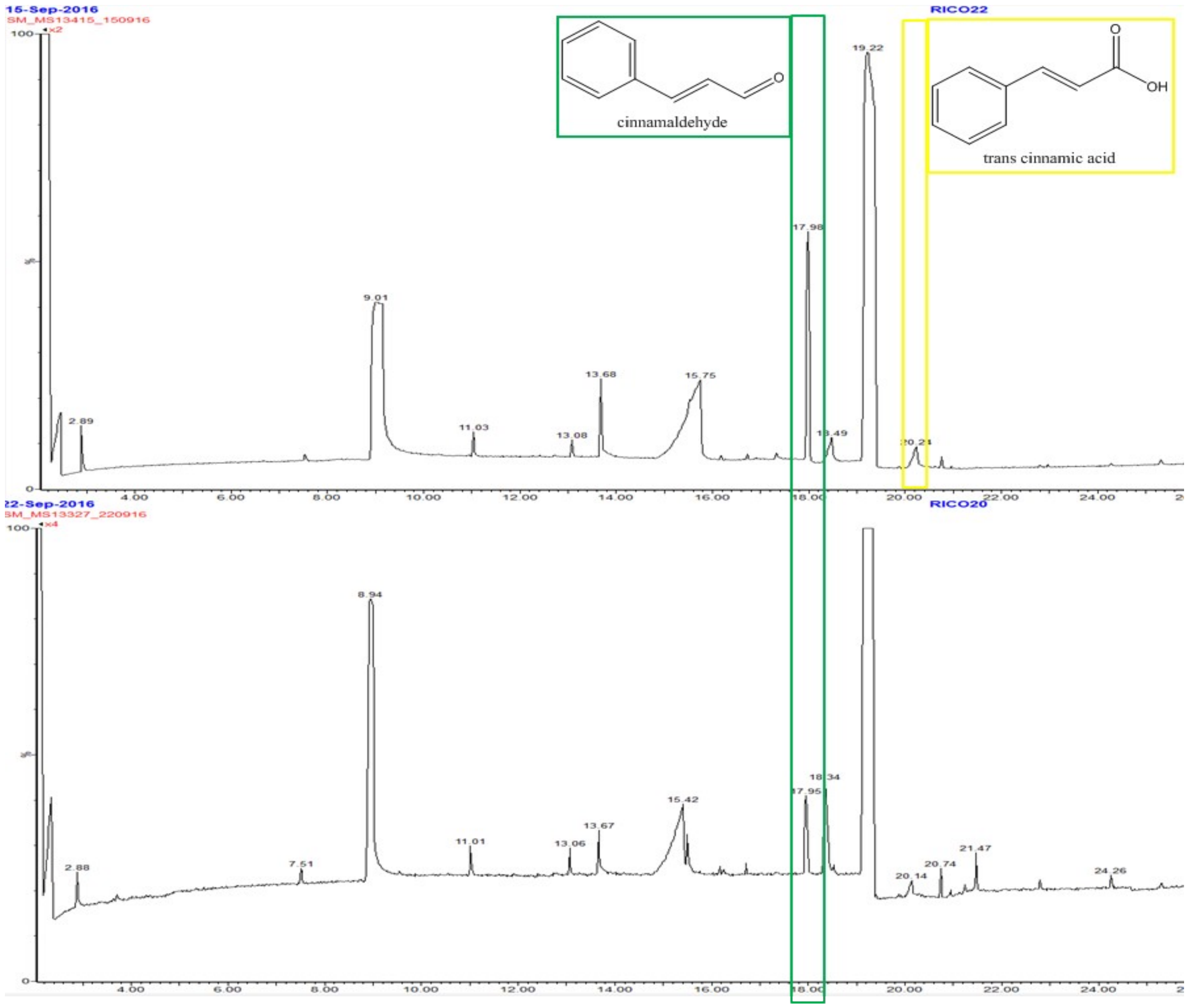

Figure S6: GC-MS spectrum of highly concentrated sample in ethyl acetate of the reactions with more oxidant ratio.

Table S4 ^{31}P-NMR chemical shifts of typical hydroxyl groups in Kraft lignin after derivatization using DP and TMDP.[1-4]

Reactive groups	***DP***	***TMDP***
Carboxyl OH	126-127.8	133-137
p-Hydroxy-phenolic OH	127.8-129	137-138.6
Guaiacyl OH	129-130.5	138.-140.2
5-substituted OH	130.5-132	140-144.5
Total condensed phenolic OH	-*	140.2-145.2
5-5' condensed OH	-*	140.2-141.4
Primary Aliphatic OH	132-133.5	145.2-151.4#
Secondary Aliphatic OH	133.5-136.5	

** 5-substituted phenolic OH (S-units and 5-condensed G-units) overlap to the aliphatic primary OH. # Aliphatic OH groups cannot be distinguish well using TMDP in most of lignin.*

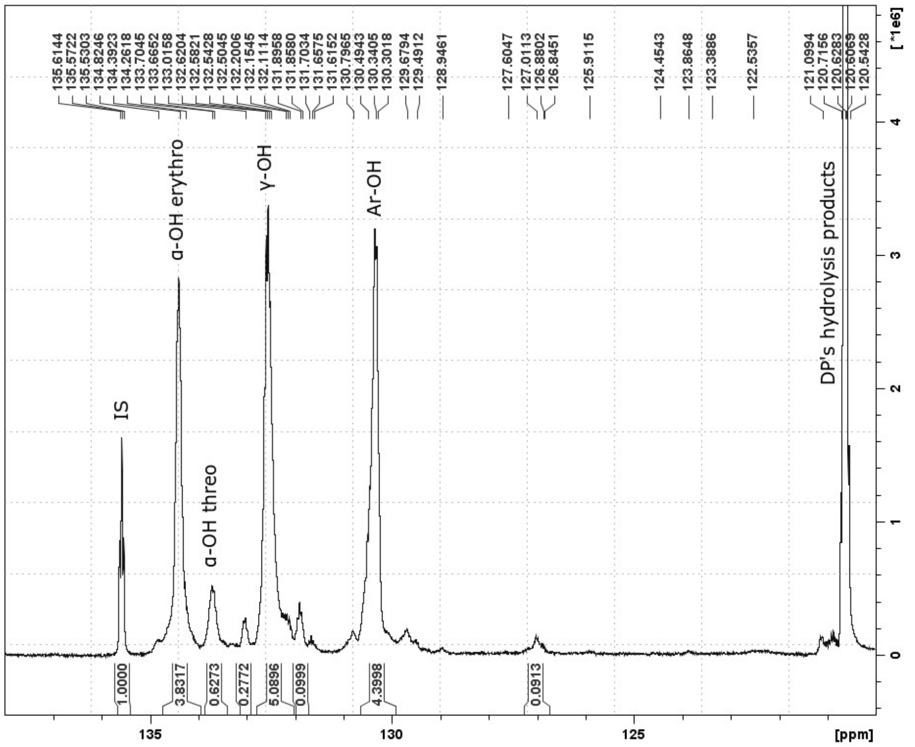

Figure S7 ^{31}P NMR spectrum of the Hexamer after derivatization using DP.

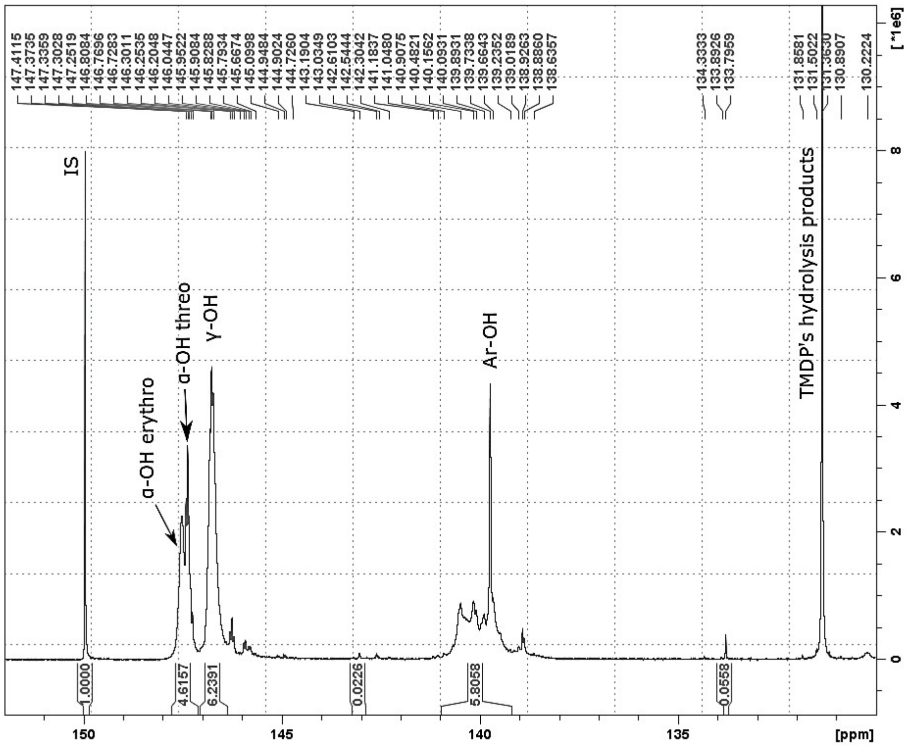

Figure S8 [31] P NMR spectrum of the Hexamer after derivatization using TMDP

Table S5 ^{31}P-NMR shifts for different internal standards after derivatization using TMDP and DP.[1-4]

Entry	***Internal Standard***	***TMDP*** ***δ ^{31}P (ppm)***	***DP*** ***δ ^{31}P (ppm)***
1	cyclohexanol	145.1	
2	cholesterol	144.9	
3	N-hydroxyphthalimide	150.7-149.9*	135.5*
4	1-hydroxy-7-azabenzotriazole	150.6	
5	N-hydroxy-5-norborene-2,3-dicarboximide	151.9	
6	N-hydroxy-1,8-napththalimide	153.6	
7	tris(2,4-di-tert-butylphenyl)phosphite	130.7	
8	Piperidine	138.7	

**Experimental value*

Table S6 Estimation of potential % conversion of different linkages present in hexamer using ^{31}P NMR

Functional OH group	*Object*	*Integration range [ppm]*	*v(F1) [ppm]*	*SM Integral [rel]*	*30 min low Integral [rel]*	*Potential conversión (%)*
Ar-OH	Integral 5	131-129.9	130.5	5.6439	0.7393	87
y-OH	Integral 4	132.85-132	132.4	5.9552	2.5371	57
a-OH threo	Integral 3	133.9-133.4	133.7	0.7127	0.0789	89
a-OH erythro	Integral 2	135-133.9	134.5	4.4476	1.2345	72
IS	Integral 1	135.7-135.45	135.6	1	1	-

Table S7 Chemical shifts of the areas δC/δH (ppm) for the inter-unit linkages present in the hexamer model compound[28-30]

Linkage/unit	***Chemical shift of the peak δC/δH (ppm)***
Methoxy groups	56/3.8
β-O-4' (Aγ)	61/3.6
β-O-4' (Aα)	73/4.98
β-O-4' (Aβ)	87/4.19
β-5'	94/5.08
5-5'	Not detected
Guaiacyl (G2)	111/7.0
Guaiacyl (G6)	121/7.07

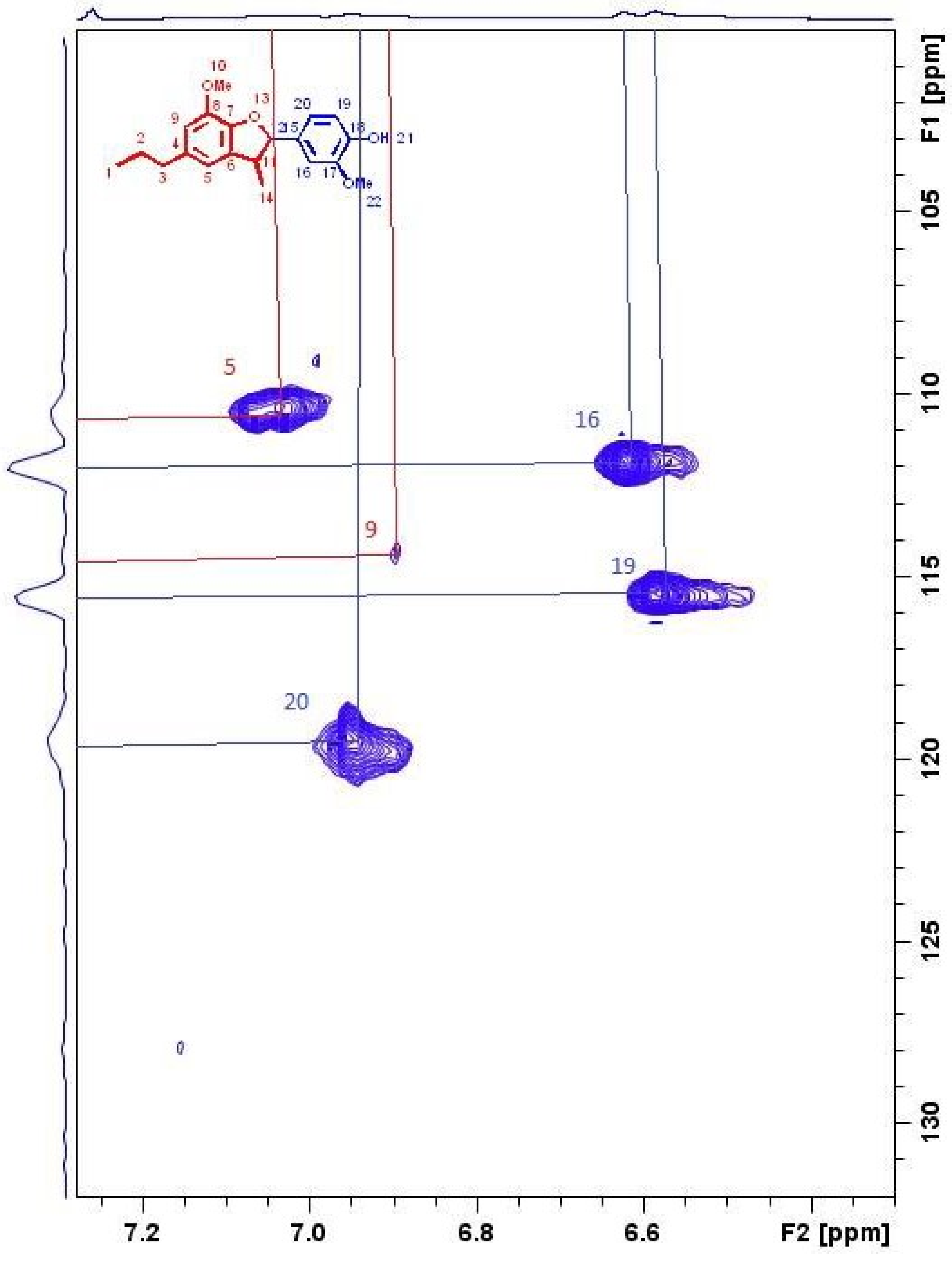

Figure S9: Partial HSQC NMR spectra of the first fraction F1

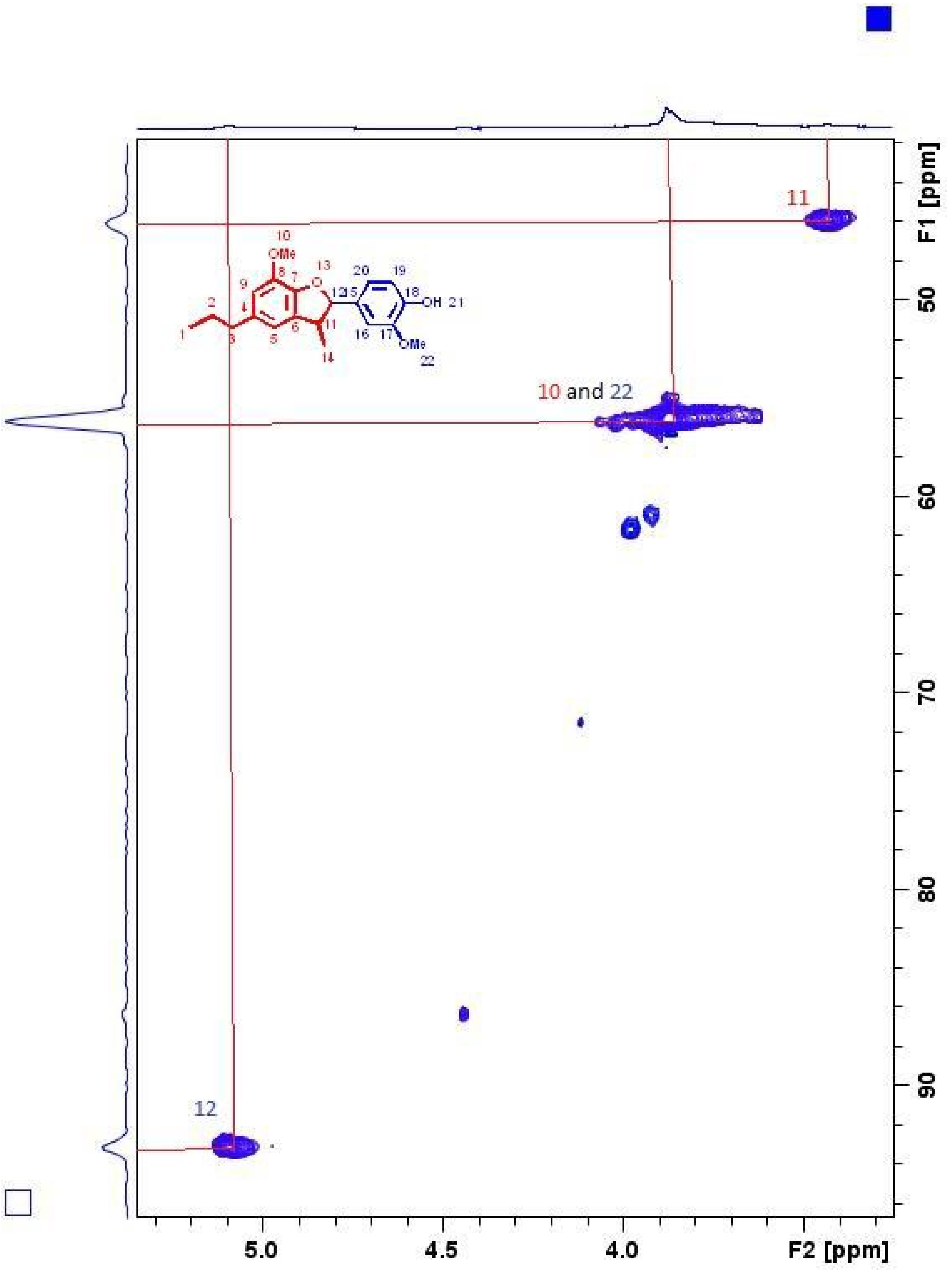

Figure S10: Partial HSQC NMR spectra of the first fraction F1

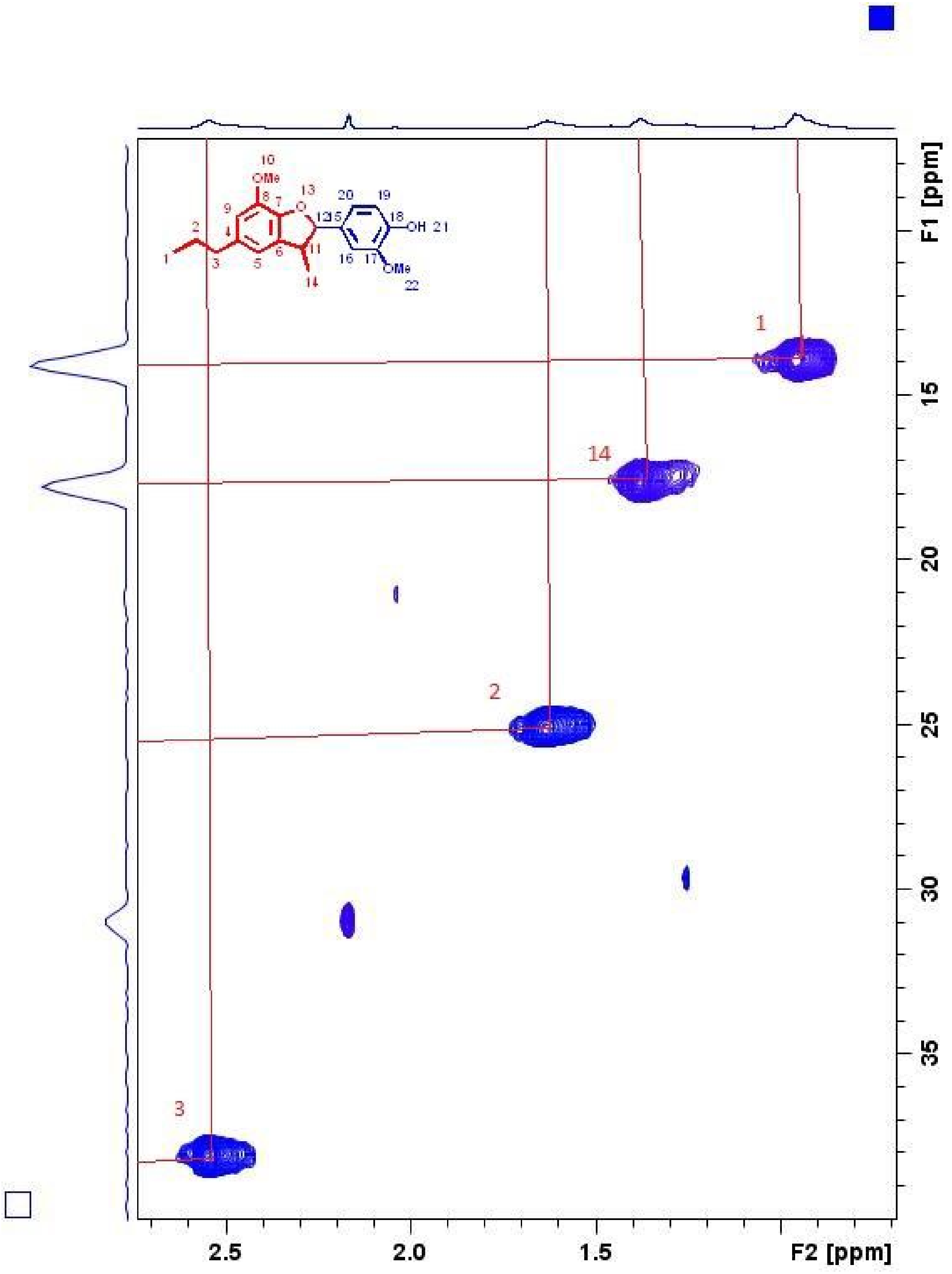

Figure S11: Partial HSQC NMR spectra of the first fraction F1

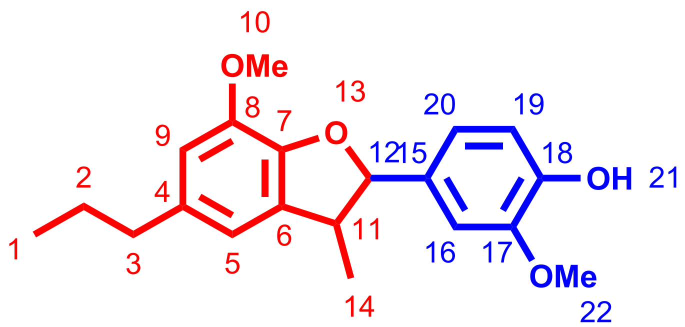

Figure S12: Structure of the β – 5 compound.

F2

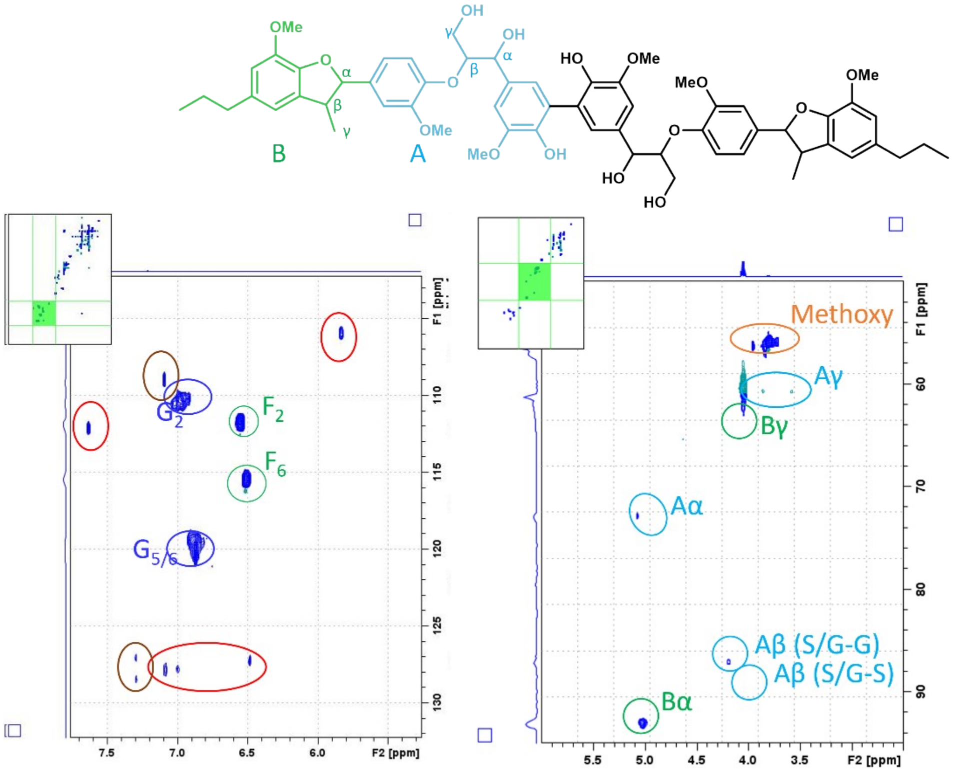

Figure S13: Partial HSQC NMR spectra of the second fraction F2

Table S8 List of potential products identified by GC-MS after the RICO of the hexamer.

Fraction	***RT (min)***	***Compound***	***Formula***	***Structure***	***GC-MS data***
F3, F4	9.7	Ethanone,1-(3-hydroxyphenyl)	$C_8H_8O_2$	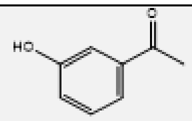	*Figure a*
F1, F2	11.05	Benzaldehyde	C_7H_6O	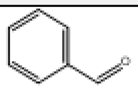	*Figure b*
F2, F3, F4	12.18	4-Methoxybenzene-1,2-diol	$C_7H_8O_3$	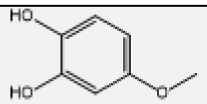	*Figure c*
F1	17.95	Cinnamaldehyde	C_9H_8O	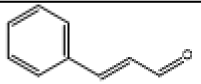	*Figure d*
F2	21.27	4,4'-Dimethylbiphenyl	$C_{14}H_{14}$	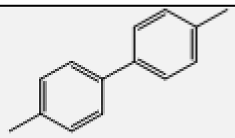	*Figure e*
F2	22.25	2-phenyl-4,5-methylenedioxybenzaldehyde	$C_{14}H_{10}O_3$	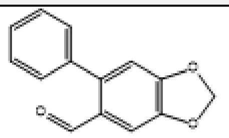	*Figure f*
F2	24.86	2-(2,6-dimethoxyphenyl)-5,6-dimethoxy-4H-chromen-4-one *[Tricin]**	$C_{19}H_{18}O_6$	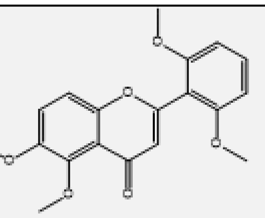	*N/A*
F1, F2, F3	34.93	2-mehoxy-4-(7-methoxy-3-methyl-5-propyl-2,3-dihydrobenzofuran-2-yl(phenol)	$C_{20}H_{24}O_4$	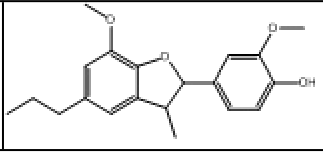	*Figure g*

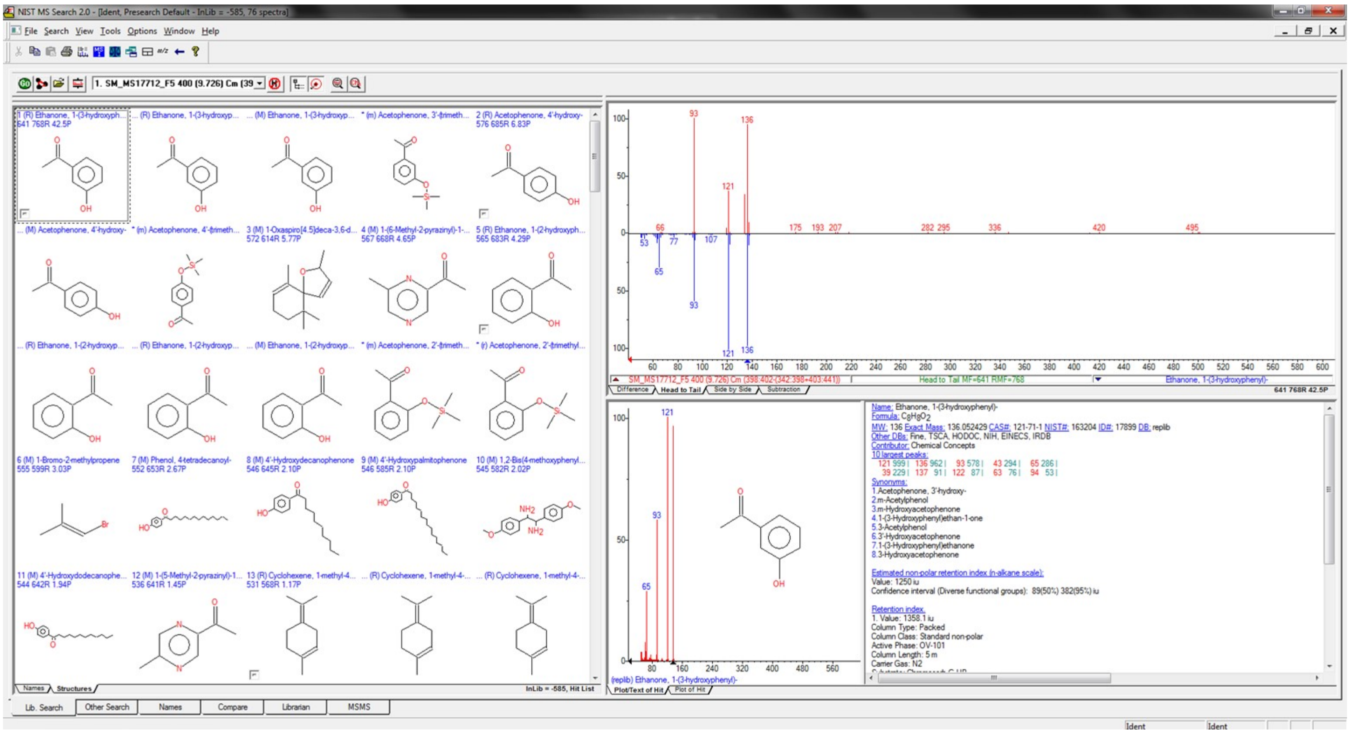

Figure a

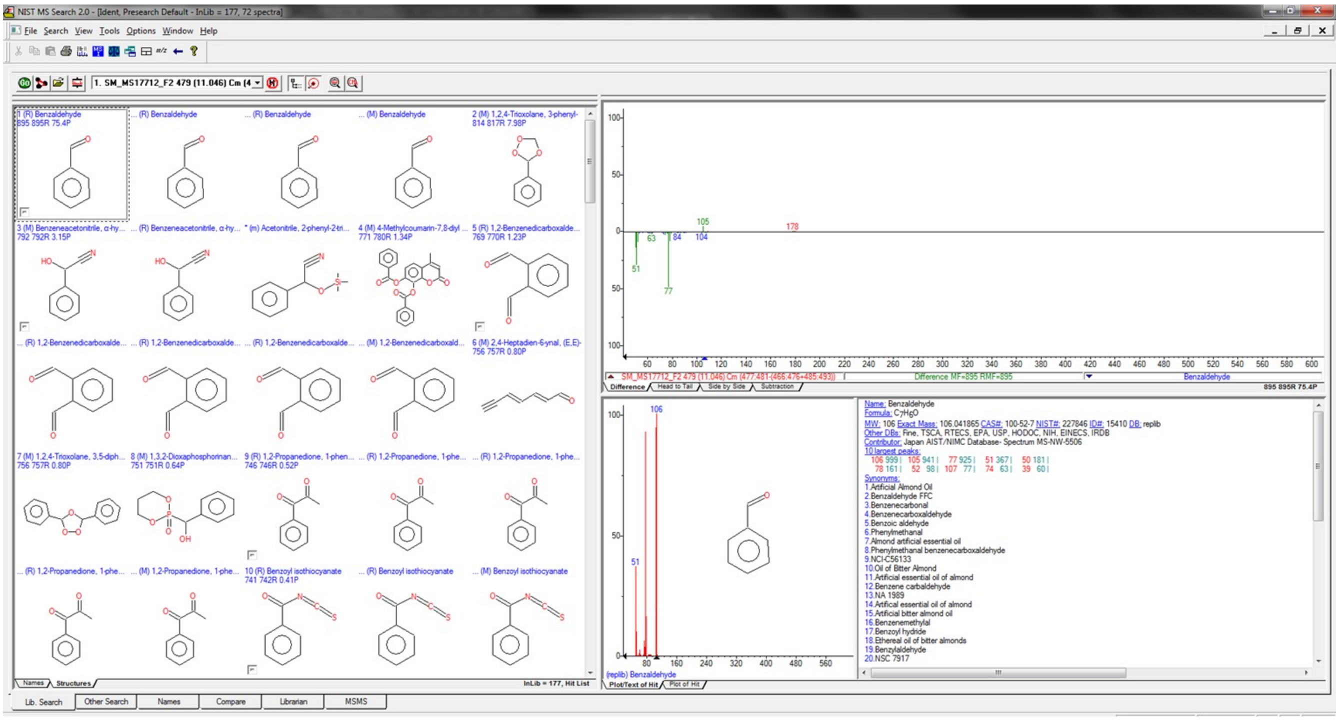

Figure b

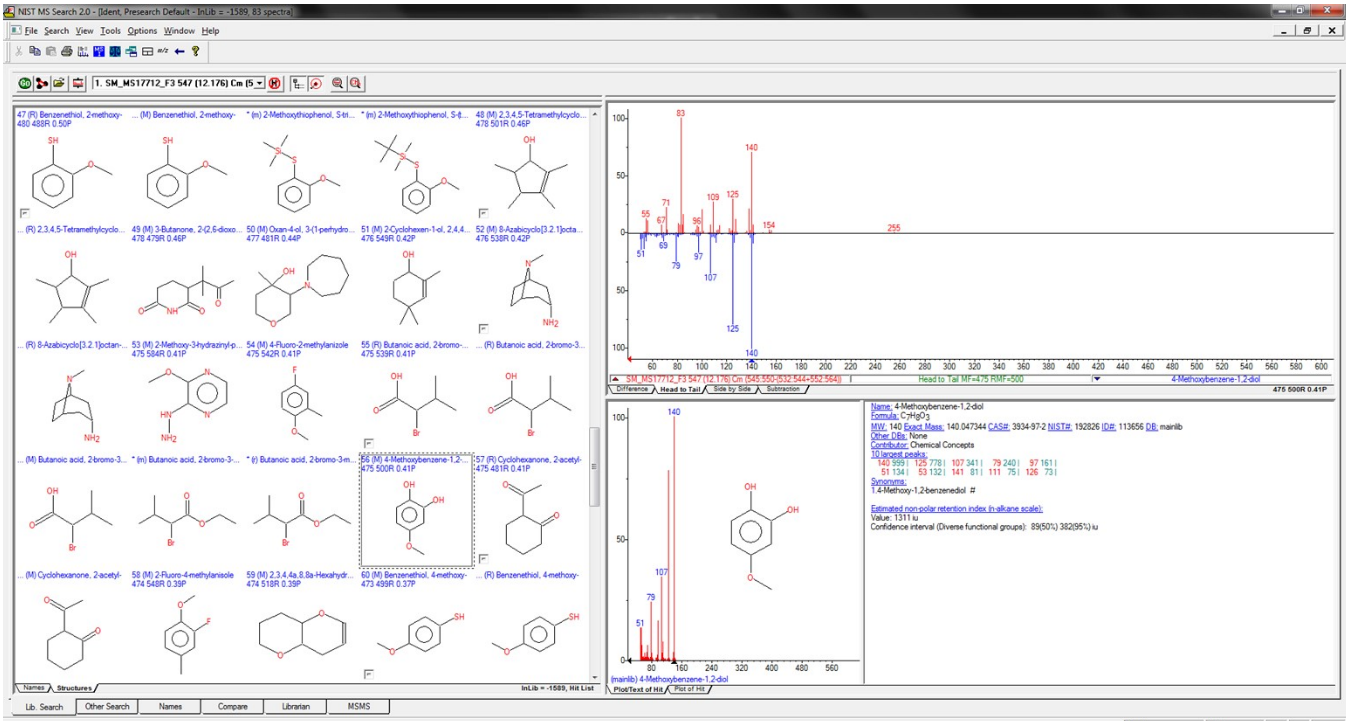

Figure c

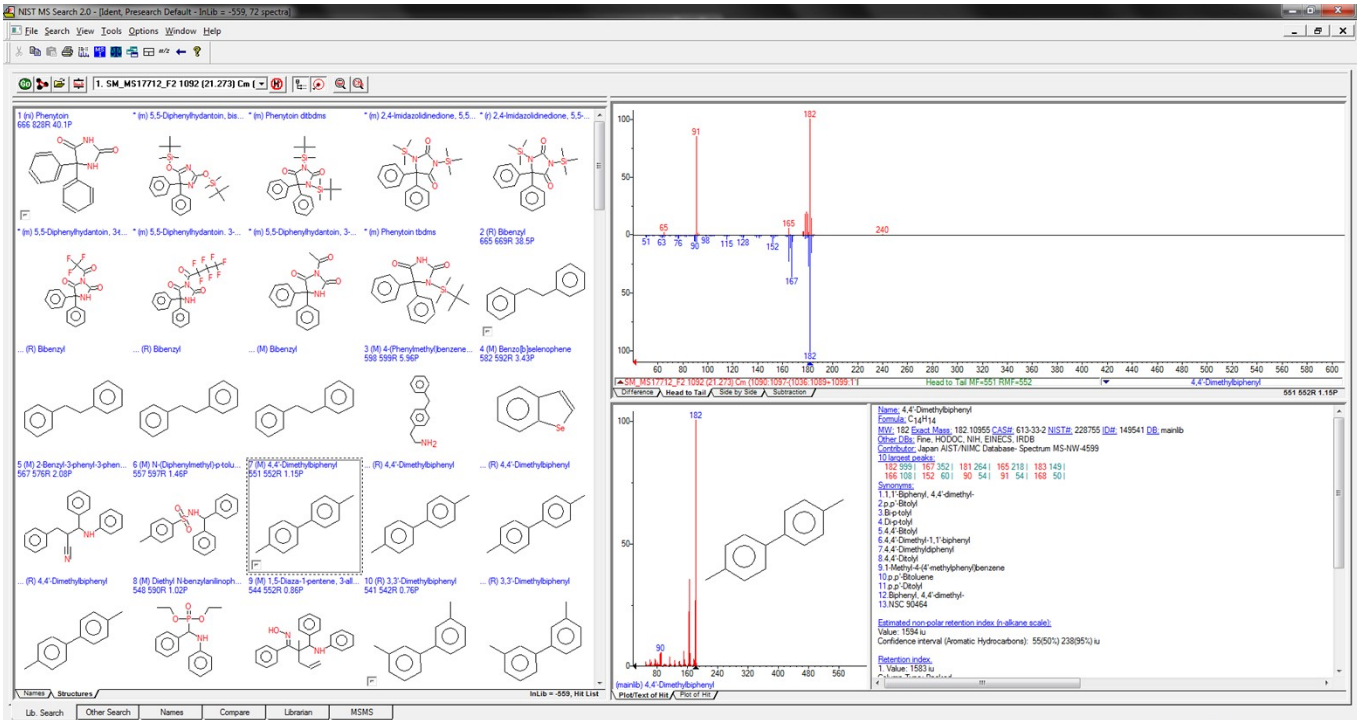

Figure d

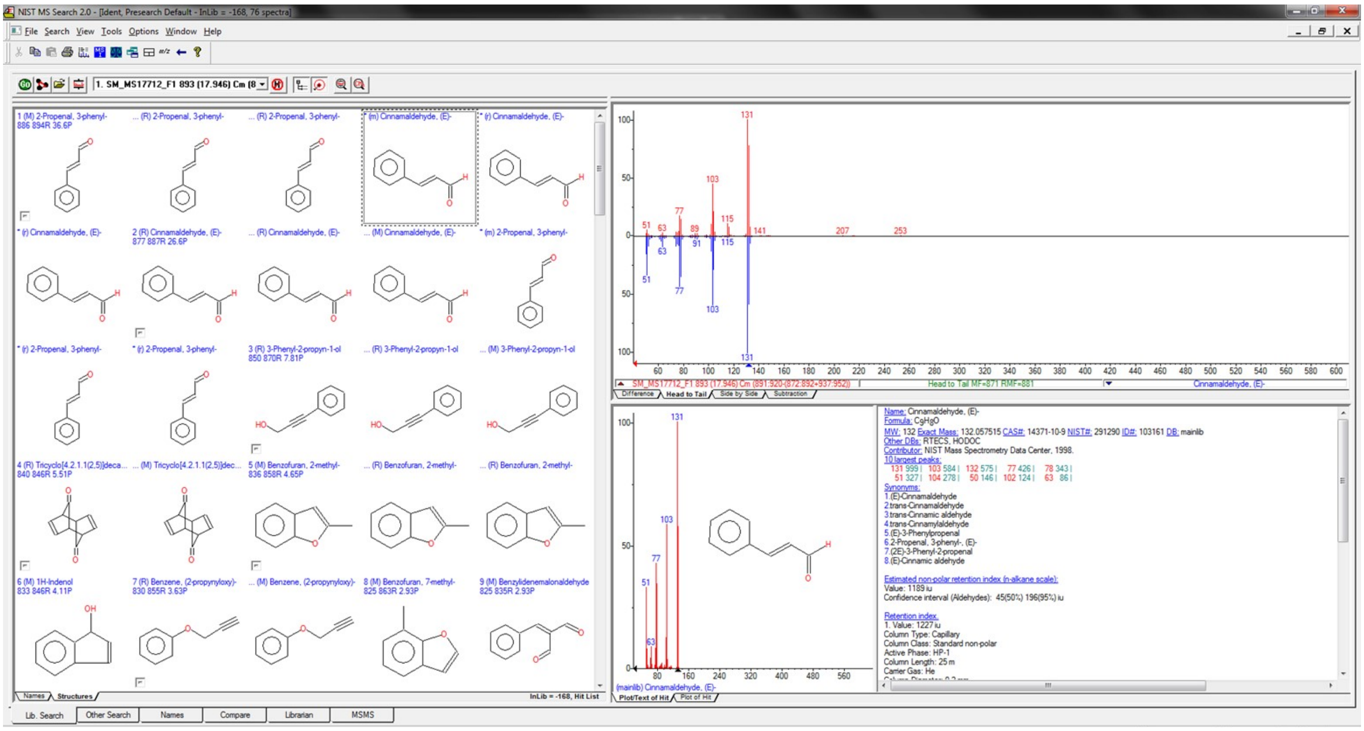

Figure e

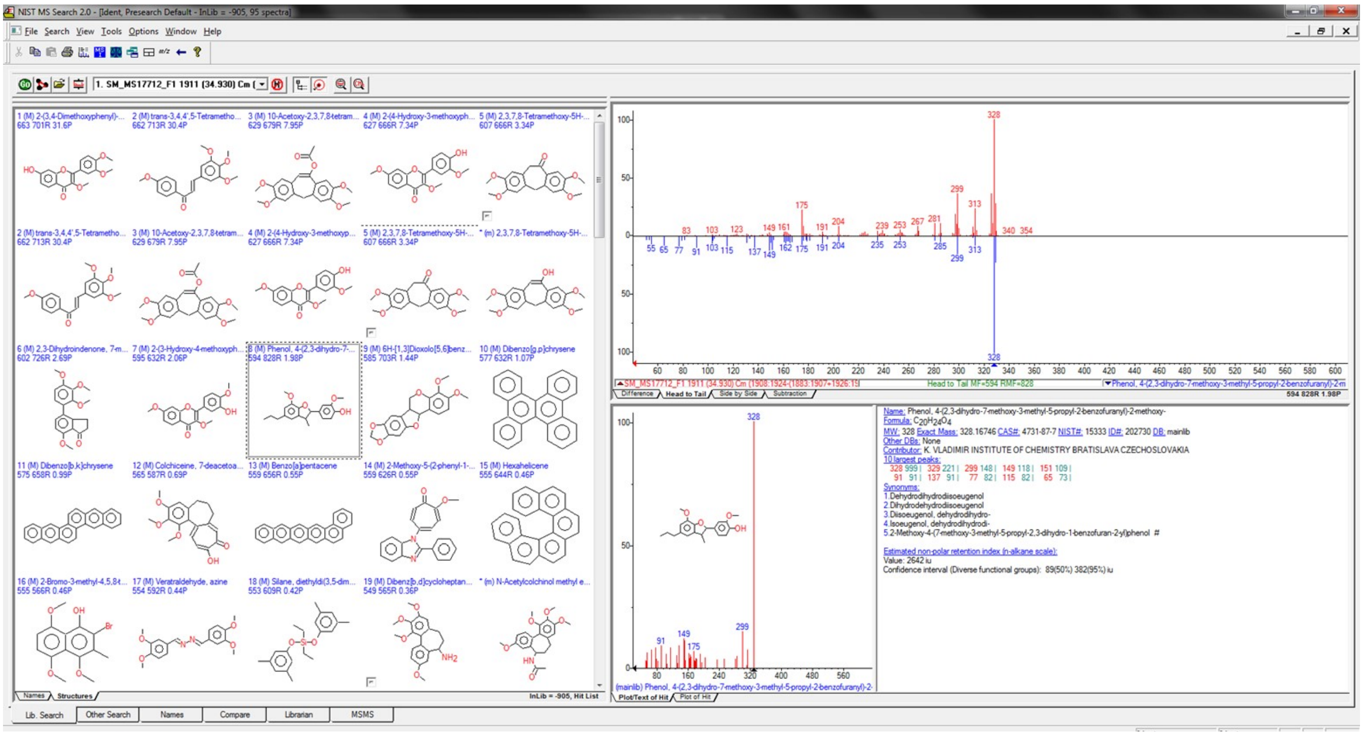

Figure f

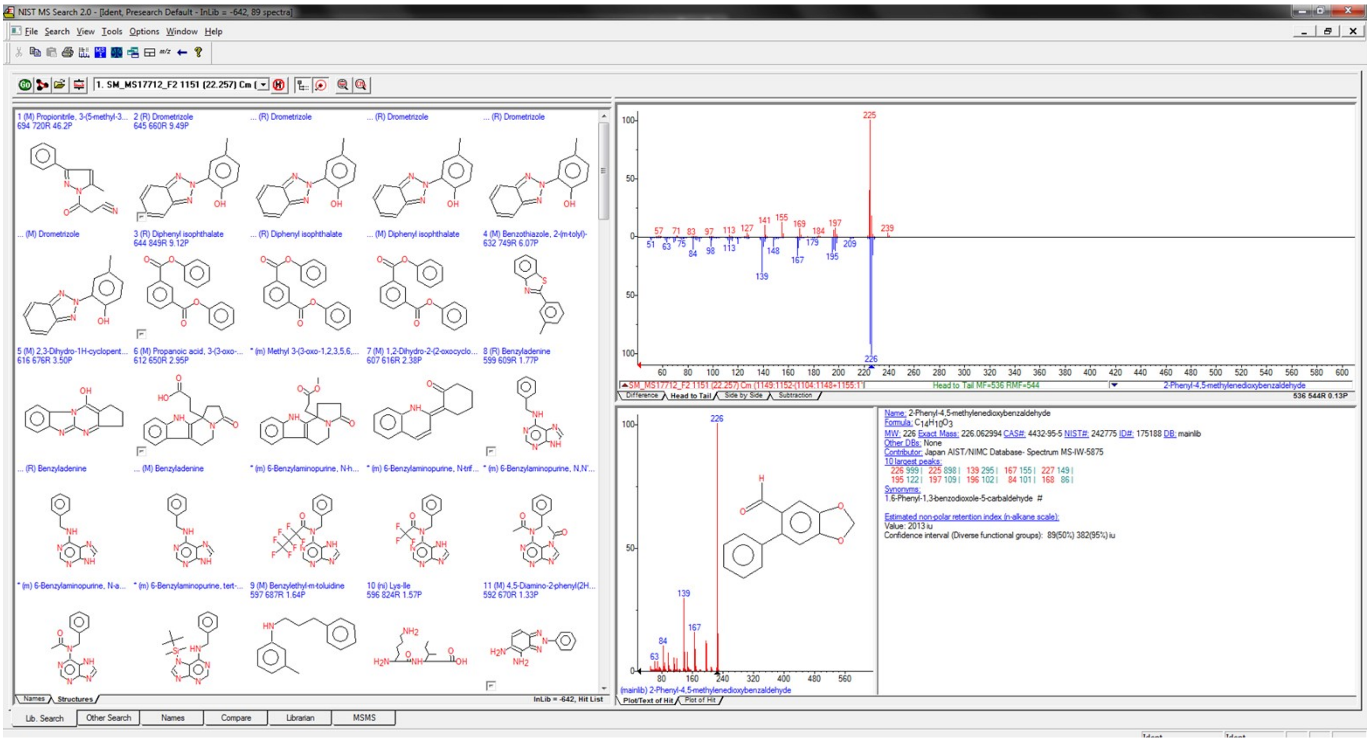

Figure g

References

1. S. Constant, H. L. J. Wienk, A. E. Frissen, P. d. Peinder, R. Boelens, D. S. van Es, R. J. H. Grisel, B. M. Weckhuysen, W. J. J. Huijgen, R. J. A. Gosselink and P. C. A. Bruijnincx, *Green Chemistry*, 2016, **18**, 2651-2665.
2. Z.-H. Jiang, D. S. Argyropoulos and A. Granata, *Magnetic Resonance in Chemistry*, 1995, **33**, 375-382.
3. Y. Pu, S. Cao and A. J. Ragauskas, *Energy & Environmental Science*, 2011, **4**, 3154-3166.
4. M. Zawadzki and A. Ragauskas, Walter de Gruyter, 2001, **55** , 283-285.
